# Supplementary material for: A cross-sectional examination of immune adaptations during pregnancy in the ECHO Cohort
Source: Front Immunol. 2026 Mar 23;17:1803430. doi: 10.3389/fimmu.2026.1803430 (PMC13050657; doi:10.3389/fimmu.2026.1803430)
Supplement: Supplementary file 1 [file DataSheet1.pdf]

Supplementary Material

1 Supplementary Figures and Tables

Supplementary Table 1. Coefficients of variation. Coefficients of variation (CVs; %) for n=69 proteins included in the analysis are reported in Supplementary Table 1. CVs were calculated within the following groups of samples, which all represented different sets of technical replicates: Control Sample Intra-Plate was calculated within each pair of control samples (technical replicates derived from a pooled plasma sample provided by the data-generating lab) on each plate (n=2 technical replicates per plate; reflects variation in technical replicates derived from a single sample within the same plate); Control Sample Inter-Plate included all control samples (technical replicates derived from a pooled plasma sample provided by the data-generating lab) across all plates (reflects variation in technical replicates derived from a single sample across all plates); Intra-Plate Study Technical Replicate was calculated within each pair of technical replicate sample pairs originating from ECHO biospecimens that were assayed on the same plate within the experiment (reflects variation in technical replicate pairs derived from multiple ECHO biospecimens assayed within the same plate); and Inter-Plate Study Technical Replicate was calculated within each pair of technical replicate samples originating from ECHO biospecimens that were assayed on different plates across the experiment (reflects variation in technical replicates derived from multiple ECHO biospecimens that were assayed across different plates). Abbreviations: CV, coefficient of variation.

Protein	UniProt ID	Olink ID	Mean Control Sample Intra-Plate CV	Control Sample Inter-Plate CV	Mean Intra-Plate Study Technical Replicate CV	Mean Inter-Plate Study Technical Replicate CV
4E-BP1	Q13541	OID00536	16.2	31.7	11.3	17.1
ADA	P00813	OID00560	9.1	14.1	12.7	9.1
AXIN1	O15169	OID00487	9.9	17.7	17.8	20.7
CASP-8	Q14790	OID00550	9.7	17.7	13.9	15.6
CCL11	P51671	OID00505	13.3	22.0	8.4	8.8
CCL19	Q99731	OID00513	14.8	26.1	13.9	11.1
CCL20	P78556	OID00556	10.8	23.0	14.2	7.6
CCL23	P55773	OID00530	14.3	27.0	12.0	10.6
CCL25	O15444	OID00551	10.1	19.2	12.1	9.1
CCL28	Q9NRJ3	OID00539	10.1	21.1	12.0	13.9
CCL3	P10147	OID00532	14.5	23.2	15.6	9.8
CCL4	P13236	OID00498	14.0	24.4	11.6	9.5
CD244	Q9BZW8	OID00477	10.2	17.1	12.4	7.9
CD40	P25942	OID00542	10.5	17.5	11.4	10.6
CD5	P06127	OID00531	9.3	17.8	12.7	7.8
CD6	P30203	OID00499	14.3	22.5	10.3	10.5
CD8A	P01732	OID05124	14.9	25.4	10.8	12.1
CDCP1	Q9H5V8	OID00476	9.5	17.7	12.1	11.2
CSF-1	P09603	OID00562	6.6	9.1	5.6	6.6
CST5	P28325	OID00491	10.8	16.2	7.8	7.3
CX3CL1	P78423	OID00552	11.3	17.9	14.7	12.1

CXCL1	P09341	OID00496	6.0	11.0	11.5	10.9
CXCL11	O14625	OID00486	13.1	25.1	11.5	10.3
CXCL5	P42830	OID00520	12.6	20.0	16.9	12.1
CXCL6	P80162	OID00534	12.4	25.6	15.0	11.6
CXCL9	Q07325	OID00490	12.6	21.7	11.7	10.2
DNER	Q8NFT8	OID01213	9.3	12.7	9.4	7.0
EN-RAGE	P80511	OID00541	18.8	44.1	20.4	10.6
FGF-19	O95750	OID00545	11.3	21.4	11.8	11.5
FGF-21	Q9NSA1	OID00512	16.9	38.9	11.9	17.2
FGF-23	Q9GZV9	OID00507	12.5	20.0	14.3	13.5
Flt3L	P49771	OID00533	10.5	20.3	10.9	8.3
HGF	P14210	OID00522	12.8	21.9	10.5	8.7
IFN-γ	P01579	OID05547	11.6	22.4	10.5	11.2
IL10	P22301	OID00528	11.7	22.4	12.9	8.9
IL-10RB	Q08334	OID00515	12.1	14.8	7.3	9.1
IL-12B	P29460	OID00523	11.3	19.7	12.4	8.0
IL-15RA	Q13261	OID00514	10.5	15.7	10.6	8.8
IL-17C	Q9P0M4	OID00483	12.1	25.3	10.9	16.0
IL18	Q14116	OID00501	13.9	23.4	12.6	11.6
IL-18R1	Q13478	OID00517	11.8	19.4	12.2	9.9
IL6	P05231	OID00482	11.5	15.7	12.7	11.6
IL7	P13232	OID00478	11.2	16.8	11.1	9.4
IL8	P10145	OID00471	13.6	23.1	13.2	9.6
LAP-TGF-β-1	P01137	OID00480	9.9	22.1	8.7	12.1
LIF-R	P42702	OID00511	12.8	20.3	9.7	12.3
MCP-1	P13500	OID00484	12.6	23.5	12.8	8.0
MCP-2	P80075	OID00549	10.0	21.3	13.0	10.0
MCP-4	Q99616	OID00504	13.7	25.7	11.8	8.2
MMP-1	P03956	OID00510	12.9	20.4	9.7	10.5
MMP-10	P09238	OID00527	13.4	19.4	10.5	6.9
NT-3	P20783	OID00554	11.6	20.2	39.9	29.3
OPG	O00300	OID00479	10.6	18.1	8.8	9.2
OSM	P13725	OID00494	10.6	20.0	9.8	11.1
PD-L1	Q9NZQ7	OID00518	14.9	19.2	11.4	11.1
SCF	P21583	OID00500	9.7	11.8	5.8	8.3
SIRT2	Q8IXJ6	OID00538	13.4	27.0	15.8	17.5
ST1A1	P50225	OID00557	8.8	18.2	16.7	20.2
STAMBP	O95630	OID00558	10.5	18.5	14.9	13.8
TGF-α	P01135	OID00503	11.6	19.9	10.4	8.7
TNF	P01375	OID05548	12.3	20.4	11.9	13.1
TNFB	P01374	OID00561	9.0	14.5	9.9	7.8
TNFRSF9	Q07011	OID00553	8.8	15.0	11.4	9.2
TNFSF14	O43557	OID00506	13.8	21.1	10.1	7.7
TRAIL	P50591	OID00488	10.4	17.6	8.3	7.9
TRANCE	O14788	OID00521	13.8	21.4	13.2	10.6
TWEAK	O43508	OID00555	12.3	24.6	14.1	9.7
uPA	P00749	OID00481	10.6	20.6	7.8	9.7
VEGFA	P15692	OID00472	11.9	21.1	12.3	8.0

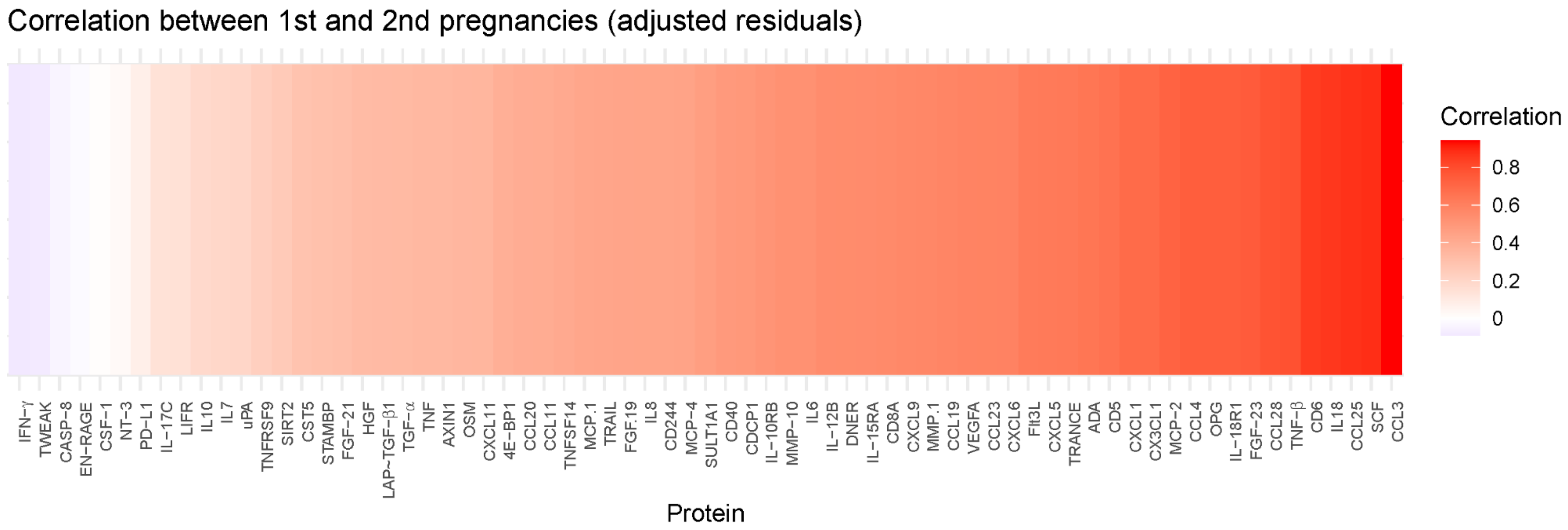

Supplementary Figure 1. Within-subject correlations in protein expression levels between subsequent pregnancies. After regressing out the effects of gestational age at blood draw, maternal age, fetal sex, and gravidity, the majority of proteins showed consistent expression levels across pregnancies within a participant. Only 4 of the 69 proteins showed correlation coefficients < 0: IFN-γ, TWEAK, CASP-8, and EN-RAGE. To evaluate within-person consistency of inflammatory protein expression across pregnancies, we identified participants with at least two pregnancies (n=30). Participants missing any protein or covariate value (gestational age at blood draw, maternal age, fetal sex, and gravidity) in either pregnancy were excluded (n=12, resulting in n=18 to be analyzed). For each participant, pregnancies were chronologically ordered based on blood draw date, and only the first two pregnancies were retained. We additionally excluded pregnancies with implausible gravidity patterns (e.g., gravidity not increasing across pregnancies). Protein values were residualized to remove effects of gestational age, maternal age, fetal sex, and gravidity using linear regression models. For each protein, residual values from pregnancy 1 and pregnancy 2 were reshaped into a wide format and pairwise Pearson correlations were computed.

Supplementary Table 2. Functional classification of the 69 inflammatory proteins from the Olink Target 96 Inflammation panel included in analyses. A list of gene names, alternative names, and biomarker names (all as defined by Olink) is provided for the 69 proteins included in our analyses. For each protein, we additionally provide a description of its general biological function based on definitions from the National Institutes of Health gene database (1) and a summary of its known or proposed roles in pregnancy-related physiological processes and/or pregnancy-associated conditions.

Gene Name	Alternative Names	Biomarker	Role in Pregnancy
CCL2	MCP1, SCYA2	C-C motif chemokine 2	Cytokine that mediates the recruitment of monocytes. Expressed by first-trimester decidual stromal cells (DSCs), promoting primary DSC proliferation and growth (2). Abnormal levels associated with adverse pregnancy outcomes (3). Increased in gestational diabetes mellitus (GMD) (4). Associated with anxiety in pregnancy (5).
CCL3	MIP1A, G0S19, SCYA3	C-C motif chemokine 3	Chemokine involved in adaptive immune response and inducible in hematopoietic cells. Increases rapidly in the first trimester when trophoblasts differentiate to extravillous trophoblasts (2).
CCL4	MIP1B, LAG1, SCYA4	C-C motif chemokine 4	Chemokine involved in adaptive immune response and inducible in hematopoietic cells. Involved in uterine receptivity and placentation in early pregnancy (6). Decreased in GDM (4). Associated with anxiety and depression in pregnancy (5).
CCL8	MCP2, SCYA10, SCYA8	C-C motif chemokine 8	Inflammatory chemokine expressed by fibroblasts and associated with Th2 immune response. In early pregnancy, CCL8 from DSCs supports proper macrophage positioning and immune tolerance. Upregulated in hypoxic states. High levels contribute to the pathogenesis of preeclampsia (7).
CCL11	SCYA11	Eotaxin	Eosinophil chemoattractant implicated in neuroinflammation and neurodegenerative disorders (8). In pregnancy, plays a pro-invasive role in decidual control of trophoblast invasion (9). Associated with depression in pregnancy (5).
CCL13	MCP4, NCC1, SCYA13	C-C motif chemokine 13	Chemotactic agent for many immune cells with an established role in rheumatologic, dermatologic, and oncologic disease. In pregnant patients with multiple sclerosis, decreased in late pregnancy and increased in the postpartum period (10).
CCL19	MIP3B, ELC, CYA19	C-C motif chemokine 19	Chemokine expressed at high levels in the thymus and lymph nodes that directs a variety of cell types to T-cell zones of lymphoid organs. Physiologically decreased in pregnancy with thymic involution (11).
CCL20	MIP3A, LARC, SCYA20	C-C motif chemokine 20	Upregulated in the plasma of patients with preeclampsia in early and late pregnancy (12). Increased levels in the lower segment myometrium found in preterm labor (13).
CCL23	MPIF1, MIP3, SCYA23	C-C motif chemokine 23	Chemoattractant of T lymphocytes, monocytes and neutrophils and angiogenesis mediator. In preeclampsia, upregulated in those with small-for-gestational age infants (14).
CCL25	TECK, SCYA25	C-C motif chemokine 25	Chemokine highly expressed by thymus and intestinal epithelium with role in mucosal immunity. Limited literature on role in pregnancy.

CCL28	SCYA28	C-C motif chemokine 28	Chemoattractant of CD4 or CD8 T cells and eosinophils involved in the mucosal homing of B and T cells. Physiologically decreased in pregnancy with thymic involution (11).
CXCL1	GRO, GRO1, GROA, MGSA, SCYB1	Growth-regulated alpha protein	Monocyte chemoattractant secreted by activated T cells and implicated in various inflammatory diseases. May stimulate angiogenesis in the first trimester via the vascular endothelial growth factor pathway (15). Increased in GDM (4).
CXCL5	ENA78, SCYB5	C-X-C motif chemokine 5	Neutrophil chemoattractant and promoter of angiogenesis. Abnormal secretion of CXL5 may be early marker of spontaneous pregnancy loss; downregulated in the villous tissue of patients with recurrent spontaneous pregnancy loss (2).
CXCL6	GCP2, SCYB6	C-X-C motif chemokine 6	Proinflammatory chemokine implicated in connective tissue metabolism and neuroinflammatory disorders. In pregnancy, inhibits trophoblast cell migration and invasion in the first trimester (16). Increased expression in perinatal mood and anxiety disorders in patients without preeclampsia (17).
CXCL9	MIG, CMK, SCYB9	C-X-C motif chemokine 9	Activated T lymphocyte chemoattractant. Increased in GDM (4). Increased in the second trimester in pregnancies resulting in preterm birth (18).
CXCL11	ITAC, SCYB11, SCYB9B	C-X-C motif chemokine 11	Activated T lymphocyte chemoattractant. Excess CXCL11 in decidua blunts peripheral natural killer (NK) cell recruitment and may contribute to shallow placentation in preeclampsia (2). Decreased in GDM (4). Increased expression in perinatal mood and anxiety disorders in patients without preeclampsia (17).
CX3CL1	FKN, NTT, SCYD1	Fractalkine	Monocyte chemoattractant with role in wide range of inflammatory, oncologic, and infectious disease. Highly expressed by cytotrophoblast decidual cells. Elevated levels associated with preterm labor and a predictive biomarker of preterm premature rupture of membranes (19). Also associated with early pregnancy loss. Elevated levels in pregnancy associated with increased maternal lifetime stress and trauma (20).
IL6	IFNB2	Interleukin-6	Cytokine with major role in inflammation and the maturation of B cells and an endogenous pyrogen, widely implicated in disease states. Proposed role in implantation and remodeling of the spiral arteries in physiologic pregnancy; abnormal expression implicated in pregnancy loss, preeclampsia, and GDM (21).
IL8	CXCL8	Interleukin-8	Proinflammatory cytokine with major role in mediating inflammatory response. Neutrophil chemoattractant and potent angiogenic factor. Implicated in physiologic pregnancy establishment and maintenance; abnormal expression implicated in pregnancy loss, preeclampsia, and GDM (21). Elevated levels associated with increased anxiety in pregnancy (5).
IL12B	NKSF2	Interleukin-12 subunit beta	Cytokine expressed by activated macrophages that induces Th1 cell development. Polymorphisms of this gene associated with preeclampsia (22).
IL17C	None	Interleukin-17C	Cytokine expressed by activated T cells that stimulates the release of TNF-alpha and IL1-beta from monocytes. Increased in preeclamptic pregnancies resulting in small-for-gestational-age infants (14). Otherwise, limited literature on role in pregnancy.
IL18	IGIF, IL1F4	Interleukin-18	Proinflammatory cytokine implicated in the injury of various organs and in cytokine storms. Limited literature on role in pregnancy.

TNF	TNFA, TNFSF2	Tumor necrosis factor	Multifunctional proinflammatory cytokine primarily secreted by macrophages. Involved in the regulation of many biologic processes and implicated in various immunologic diseases. TNF-alpha influences hormone synthesis, placental architecture, and embryonic development in physiologic pregnancy, and increased levels are associated with poor pregnancy outcomes including early pregnancy loss and preeclampsia (23).
TNFSF10	TRAIL, APO2L	Tumor necrosis factor ligand superfamily member 10	Cytokine that preferentially induces apoptosis in transformed and tumor cells. Expressed at the maternal–fetal interface to induce apoptosis of lymphocytes to maintain immune tolerance. Induces apoptosis of vascular smooth muscle cells for spiral artery remodeling in physiologic pregnancy. Decreased levels in preeclampsia (24).
TNFSF12	TWEAK, APO3L, DRLG	Tumor necrosis factor ligand superfamily member 12	Cytokine that induces apoptosis via multiple pathways and regulates angiogenesis. Expressed in the placenta may contribute to cell death and immune regulation at the maternal–fetal interface. Low levels associated with preeclampsia and GDM (25,26).
TNFSF14	LIGHT, HVEML	Tumor necrosis factor ligand superfamily member 14	Herpesvirus entry mediator that also stimulates T cell proliferation and apoptosis. Expressed in the placenta and may contribute to cell death and immune regulation at the maternal–fetal interface. Elevated in preeclampsia (27).
IFNG	None	Interferon gamma	Cytokine secreted by innate and adaptive immune system cells that triggers cellular response to viral and microbial infections. In physiologic pregnancy, mediates uterine spiral artery remodeling; aberrant expression leads to embryotoxicity and abnormal placental development (28).
S100A12	EN-RAGE	Protein S100-A12	Calcium-binding protein that regulates cellular processes including cell cycle progression and may mediate neutrophil function. Increased levels in preeclampsia, chorioamnioniitis, and polycystic ovary syndrome (29).
IL10	None	Interleukin-10	Cytokine with many effects on inflammation and immunoregulation. Plays a major anti-inflammatory and immunoregulatory role in pregnancy, maintaining immune tolerance. Decreased levels associated with adverse pregnancy complications (preterm birth, miscarriage, fetal growth restriction) and preeclampsia (30).
TGFB1 (LAP form)	TGFB	Transforming growth factor-β1 proprotein	Pleiotropic cytokine that regulates cell proliferation and growth; predominantly anti-inflammatory in effect and frequently elevated in tumor cells. As immunosuppressive, regulates several aspects of pregnancy, such as trophoblast uterine invasiveness. Elevated in preeclampsia (31). Altered expression may contribute to the pathogenesis of recurrent pregnancy loss (32).
LTA	TNFB, TNFSF1	Lymphotoxin-alpha	Cytokine produced by lymphocytes as part of tumor necrosis factor protein family. Proinflammatory and pro-apoptotic; mediates various inflammatory, immunostimulatory, and antiviral responses. Implicated in preeclampsia and pregnancy loss, with polymorphisms associated with recurrent pregnancy loss (33).
VEGFA	VEGF	Vascular endothelial growth factor A	Induces proliferation and migration of vascular endothelial cells. Essential for physiologic and pathologic angiogenesis. In pregnancy, supports placental formation, spiral artery remodeling,

			and embryo implantation and plays a role in immune regulation. Dysregulated in preeclampsia, early pregnancy loss, recurrent miscarriage, and fetal growth restriction (34).
HGF	HGF	Hepatocyte growth factor	Pleiotropic factor that regulates cell growth, motility, morphogenesis, and repair in various cell types. In pregnancy, stimulates trophoblast invasion and contributes to placental vascular development as well as fetal organ development. Reduced in preeclampsia and intrauterine growth restriction (35).
FGF19	None	Fibroblast growth factor 19	Metabolic hormone with significant role in bile acid metabolism and growth factor involved in embryonic nervous and skeletal system development (36). Reduced levels in insulin resistance, including GDM. Positively correlated with birth weight in female infants (37).
FGF21	None	Fibroblast growth factor 21	Metabolic hormone that promotes insulin sensitivity. Rises in the third trimester as metabolic demands increase. Elevated in GDM and preeclampsia, likely to compensate for insulin resistance (38).
FGF23	HYPF	Fibroblast growth factor 23	Bone-derived protein that regulates renal phosphate and vitamin D homeostasis. Plays a role in the pathogenesis of gestational hypertension and fetal growth disorders; potential biomarker for fetal growth (39).
KITLG	Stem cell factor (SCF), MGF	Kit ligand	Pleiotropic growth factor involved in stem cell differentiation and fetal germ cell development, neural cell development, and hematopoiesis. Expressed by the placental villous core and may regulate trophoblast proliferation (40). Activates primordial follicles and plays role in fertility (41).
CSF1	None	Macrophage colony-stimulating factor (M-CSF) 1	Cytokine involved in macrophage production, differentiation, and function. In pregnancy, regulates placental development function. Dysregulation may contribute to preeclampsia and gestational hypertension (42).
LIFR	None	Leukemia inhibitory factor receptor	Receptor for leukemia inhibitory factor, a cytokine in the IL-6 family involved in fetal and adult cellular differentiation and proliferation. Regulates endometrial receptivity and implantation, including trophoblast cells and macrophage responses to inflammatory cytokines (43).
OSM	None	Oncostatin-M	IL-6 family cytokine and growth regulator that inhibits tumor cell line proliferation and regulates the production of various cytokines. Contributes to embryo implantation and decidualization and stimulates release of human chorionic gonadotrophin (44).
TGFA	None	Protransforming growth factor-α	Growth factor and ligand for epidermal growth factor receptor associated with multiple cancers. In physiologic pregnancy, serves as a major anti-inflammatory cytokine and stimulates placental mitosis. Elevated in cord blood of neonates with intrauterine growth restriction (45).
NTF3	None	Neurotrophin-3	Neurotrophin that may influence embryonic neural development and contribute to angiogenesis during embryogenesis; not significantly associated with fetal growth disorders (46).
PLAU	None	Urokinase-type plasminogen activator (uPA)	Serine protease that converts plasminogen to plasmin, a key step in fibrinolysis. Regulates trophoblast invasion and migration in early placentation (47).

MMP1	CLG	Interstitial collagenase	Protease that breaks down interstitial collagens (type I and III). A physiologic constituent of amniotic fluid that increases with gestational age; increased in premature preterm rupture of membranes (48).
MMP10	STMY2	Stromelysin-2	Protease that breaks down various extracellular matrix proteins including collagens, fibronectin, elastin, and laminin. Widely expressed at the maternal–fetal interface in pregnancy and stimulated by interleukin 1β (49). Reduced levels in preeclampsia (50).
TNFSF11	TRANCE, RANKL, OPGL	Tumor necrosis factor ligand superfamily member 11	Ligand for osteoprotegerin with major role in osteoclast differentiation and activation that also serves as a dendritic cell survival factor involved in regulating cell-mediated immunity. Elevated levels observed postpartum (51). Polymorphisms associated with preeclampsia (52).
TNFRSF11B	OPG, OCIF	Tumor necrosis factor receptor superfamily member 11B	Osteoprotegerin ligand and decoy receptor secreted by osteoblasts that downregulates bone reabsorption. May contribute to the regulation of bone turnover in pregnancy, but the literature is limited (53).
CD274	B7H1, PDCD1L1, PDCD1LG1, PDL1	Programmed cell death 1 ligand 1	Immune inhibitory receptor ligand that inhibits T-cell activation and cytokine production. Physiologically, prevents the development of autoimmunity; in tumors, can allow immune evasion. In pregnancy, expressed by the placenta, especially by syncytiotrophoblasts and cytotrophoblasts, to maintain immune tolerance, preventing fetal rejection (54). Per animal models, signaling of this pathway may be altered in pregnancy loss (55). Expression is decreased in preterm labor (56).
CD244	2B4	NK cell receptor 2B4	Cell surface receptor primarily expressed by NK cells that mediates cytotoxicity outside of the major histocompatibility complex. In pregnancy, expression in decidual NK cells contributes to immunoregulation and fetal tolerance (57). Reduced expression found in recurrent pregnancy loss (58).
CD40	TNFRSF5	Tumor necrosis factor receptor superfamily member 5	Receptor on antigen-presenting cells that serves as a key immune checkpoint, mediating T cell-dependent immunoglobulin class switching, memory B cell development, and germinal center formation. Per animal models, may play a role in the establishment of pregnancy through regulating endometrial endothelial cell function during implantation (59). Its ligand, soluble CL40L, is increased in preeclampsia and other hypertensive disorders of pregnancy (60).
CD5	LEU1	T-cell surface glycoprotein CD5	T cell, B1-a cell, and B cell chronic lymphocytic leukemia cell receptor that modulates T and B cell signaling. CD5+CD1d+ B regulatory cells are decreased in healthy pregnancy (61), while CD19+ CD5+ B cells are reduced in preeclampsia (62); however, literature on the receptor's specific role in pregnancy is limited.
CD6	None	T-cell differentiation antigen CD6	T cell membrane protein that contributes to T cell activation. Limited literature in pregnancy.
CD8A	MAL	T-cell surface glycoprotein CD8 alpha chain	Receptor found on most cytotoxic T cells that acts as a co-receptor for antigens displayed by antigen-presenting cells with major histocompatibility complex class 1 molecules. Used as a

			marker for CD8+ T-cells, which are abundant at the maternal–fetal interface; however, the literature on the receptor's specific role in pregnancy is limited (63).
CDCP1	TRASK	CUB domain-containing protein 1	Transmembrane protein with role in regulating cellular events during tumor invasion and metastasis. Expressed by a subset of trophoblasts, but literature is limited in pregnancy (64).
IL10RA	IL10R	Interleukin-10 receptor subunit alpha	Protein that mediates interleukin 10 signaling and inhibits proinflammatory cytokines. Coexpression with IL10RB required for IL-10 signaling. IL-10R is increased in the early pregnant endometrium and may act as an anti-inflammatory paracrine mediator at the maternal-fetal interface (65).
IL10RB	CRFB4, D21S58, D21S66	Interleukin-10 receptor subunit beta	See above; coexpression with IL10RA required for IL-10 signaling.
IL15RA	None	Interleukin-15 receptor subunit alpha	Cytokine receptor that binds interleukin 15 with high affinity and specificity. IL15RA is expressed by the placenta and by decidual NK cells; IL-15 stimulates in vitro proliferation of decidual NK cells without transforming them to be cytotoxic to trophoblasts (66). IL-15 is increased in preeclampsia and decreased in implantation failure (67).
IL18R1	IL1RRP	Interleukin-18 receptor 1	Cytokine receptor in the interleukin 1 receptor family that specifically binds interleukin 18 and is expressed by the endometrium and by villous trophoblasts. In early pregnancy, IL18 may regulate the cytotoxicity of NK cells. IL18 is also elevated during labor and in various pregnancy complications (68).
TNFRSF9	CD137, ILA	Tumor necrosis factor receptor superfamily member 9	Receptor in the TNF-receptor family that contributes to T cell development, survival, and clonal expansion as well as proliferation of peripheral monocytes. In pregnancy, expression increases in the uterus during implantation in animal models, but its function is unknown (69).
AXIN1	AXIN	Axis inhibition protein 1	Cytoplasmic protein that can induce apoptosis as an antagonist of the WNT/β-catenin signaling pathway; mutations associated with various cancers. Higher expression found in intrauterine growth restriction placentae (70). Lower levels in late pregnancy associated with postpartum depression (51).
CST5	None	Cystatin D	Type 2 cystatin with expression restricted to the submandibular and parotid glands (71). No known role in pregnancy.
DNER	BET	Delta and Notch-like EGF-related receptor	Protein expressed by the developing and mature central nervous system (72). No known role in pregnancy.
EIF4EBP1	4E-BP1	Eukaryotic translation initiation factor 4E-binding protein 1	Translation repressor protein that binds to eukaryotic translation initiation factor 4E (eIF4E) to inhibit protein translation. In animal models, plays a role in spindle formation in meiosis and in the transition of maternal to embryonic control of development (73). Otherwise, literature is limited in pregnancy.

SIRT2	SIR2L, SIR2L2	NAD-dependent protein deacetylase sirtuin-2	Protein involved in carbohydrate metabolism and the cell cycle with emerging role in female fertility (74). Found in placental syncytiotrophoblasts and downregulated in preeclampsia (75).
STAMBP	AMSH	STAM-binding protein	Deubiquitinating enzyme with a defective form leading to microcephaly-capillary malformation syndrome. Lower levels in late pregnancy associated with postpartum depression (76).
SULT1A1	STP, STP1	Sulfotransferase 1A1	Sulfate conjugation enzyme functional in the placenta, with substrates that include endogenous hormones (estradiol, thyroid hormones) and endogenous substances such as medicinal drugs and environmental chemicals, with likely role in mediating fetal xenobiotic exposure (77). Lower levels in late pregnancy associated with postpartum depression (76).
CASP8	MCH5	Caspase-8	Apoptotic initiation factor involved in Fas-induced programmed cell death and other apoptotic pathways. Low expression in physiologic pregnancy but elevated in preterm labor, preeclampsia, and intrauterine growth restriction (78).
ADA	ADA1	Adenosine deaminase	Enzyme in the purine catabolic pathway essential for lymphoid cell differentiation, defective in severe combined immunodeficiency disease. Increased in recurrent pregnancy loss, preeclampsia, and hyperemesis gravidarum (79). Lower levels in late pregnancy associated with postpartum depression (76).

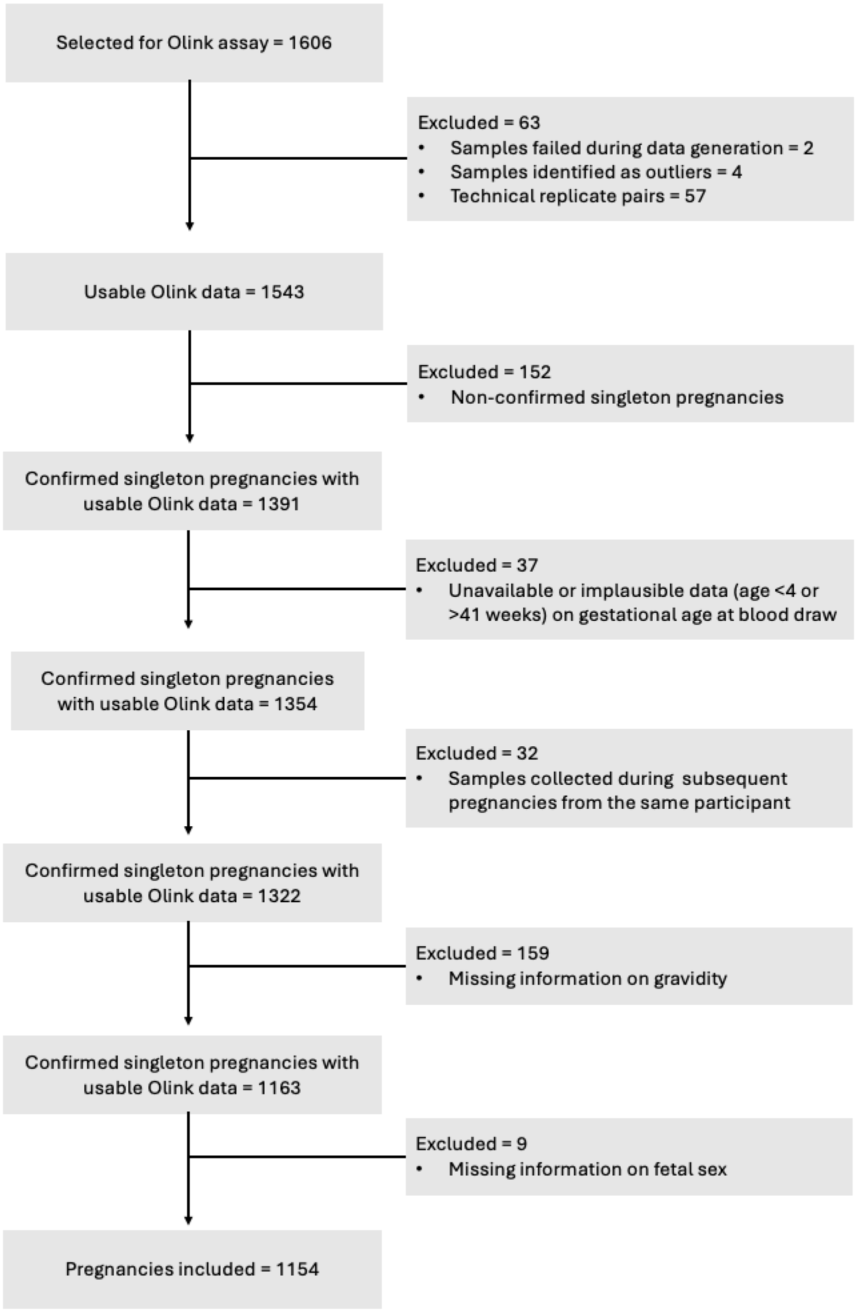

Supplementary Figure 2. Flow chart depicting each stage of participant exclusions. A total of 1154 pregnancies were included in the final analysis.

Supplementary Table 3. Trimester differences in protein expression levels. To identify markers that differed by trimester, we fit separate linear mixed-effects regression models for each protein, predicting expression level from trimester. Models controlled for maternal age at blood draw, gravidity, and infant sex and contained a random intercept for site to account for site-level heterogeneity. Because sample sizes differed across trimesters, we used linear mixed-effects regression with restricted maximum likelihood estimation, which provides valid estimates under unbalanced designs. Overall trimester effects were evaluated using Type III sums of squares, yielding an omnibus F-test for the null hypothesis that mean expression levels were equal across trimesters. Multiple testing was addressed by controlling the false discovery rate (FDR) at 5% across all proteins using the Benjamini–Hochberg procedure. To visualize the direction of trimester effects, we extracted the fixed-effect coefficients for the third trimester contrasts (relative to the first trimester) and ordered proteins by the sign and magnitude of these estimates. When modeling trimester as a categorical predictor, we observed widespread differences in protein expression across pregnancy. Mixed-effects regression models identified 53 out of 69 inflammatory proteins that showed significant differences after correcting for multiple comparisons with an FDR of 5% (Supplementary Table 3; Supplementary Figure 3). These trimester-related changes spanned multiple functional groups, including chemokines (e.g., CCL28, CCL11), growth factors (e.g., LIFR, uPA), pro-inflammatory cytokines (e.g., TWEAK), and other regulatory or signaling proteins (e.g., PD-L1, CD8A). Abbreviations: AIC, Akaike information criterion; CI, confidence interval; FDR, false discovery rate.

Protein	T1–T2 β estimate	T1–T2 t statistic	T1–T2 p	T1–T2 95% CI	T1–T3 B estimate	T1–T3 t statistic	T1–T3 p	T1–T3 95% CI	AIC	Omnibus F	Omnibus p (uncorrected)	Omnibus p (FDR-adjusted)
PD-L1	1.129	17.554	2.84E-61	[1.003, 1.255]	1.696	25.396	8.55E-111	[1.565, 1.827]	1767.670	322.839	0.000	4.73E-109
LIF-R	1.018	15.161	1.86E-47	[0.887, 1.150]	1.478	21.383	1.01E-80	[1.342, 1.613]	1866.333	229.301	0.000	1.50E-80
TWEAK	-0.653	-14.216	2.30E-42	[-0.743, -0.563]	-0.750	-15.919	2.23E-49	[-0.842, -0.658]	994.186	138.191	0.000	8.19E-52
uPA	0.168	3.528	4.36E-04	[0.074, 0.261]	0.656	13.213	3.66E-37	[0.559, 0.754]	1076.232	111.976	0.000	6.85E-44
CDCP1	0.289	4.009	6.50E-05	[0.148, 0.430]	0.688	11.478	6.02E-29	[0.571, 0.806]	2039.151	89.721	0.000	8.67E-36
CCL28	1.200	10.453	1.76E-24	[0.975, 1.425]	1.937	18.530	4.25E-31	[1.732, 2.142]	3101.498	178.804	0.000	1.21E-32
LAP-TGF-β-1	0.093	1.463	1.44E-01	[-0.032, 0.219]	0.666	9.989	1.45E-22	[0.536, 0.797]	1753.690	73.885	0.000	6.71E-30
IL-10RB	0.222	6.577	7.26E-11	[0.156, 0.288]	0.403	11.643	6.05E-29	[0.335, 0.471]	284.321	69.107	0.000	9.27E-28
CD8A	-0.693	-7.163	1.41E-12	[-0.882, -0.503]	-1.135	-11.387	4.28E-28	[-1.331, -0.940]	2702.830	65.014	0.000	2.21E-26

TRANCE	-0.504	-7.160	1.44E-12	[-0.642, -0.366]	-0.773	-11.002	2.60E-24	[-0.911, -0.635]	1972.979	60.600	0.000	1.79E-23
CCL11	-0.531	-8.857	3.08E-18	[-0.648, -0.413]	-0.627	-10.629	2.32E-21	[-0.742, -0.511]	1604.189	58.927	0.000	1.03E-21
MCP-4	-0.721	-7.553	8.68E-14	[-0.908, -0.534]	-0.951	-9.588	6.39E-21	[-1.146, -0.757]	2674.359	47.192	0.000	1.25E-19
IL-12B	-0.605	-8.070	1.77E-15	[-0.752, -0.458]	-0.673	-8.901	1.46E-17	[-0.822, -0.525]	2116.587	43.546	0.000	1.04E-17
OPG	0.260	3.563	3.82E-04	[0.117, 0.403]	0.632	8.546	1.77E-16	[0.487, 0.777]	2055.478	40.879	0.000	8.88E-17
DNER	-0.238	-6.977	5.08E-12	[-0.305, -0.171]	-0.264	-7.636	1.16E-13	[-0.331, -0.196]	312.901	32.237	0.000	1.92E-13
FGF-23	0.422	2.158	3.12E-02	[0.039, 0.805]	1.402	7.325	1.02E-11	[1.027, 1.778]	4318.647	34.404	0.000	2.59E-13
CCL23	-0.390	-6.077	1.67E-09	[-0.515, -0.264]	-0.489	-7.387	3.48E-13	[-0.618, -0.359]	1759.821	28.464	0.000	3.83E-12
MCP-2	-0.635	-6.769	2.07E-11	[-0.819, -0.451]	-0.663	-6.987	8.74E-12	[-0.849, -0.477]	2631.874	28.308	0.000	5.74E-12
TNFSF14	-0.465	-6.233	6.41E-10	[-0.612, -0.319]	-0.530	-6.814	1.56E-11	[-0.683, -0.378]	2111.311	25.812	0.000	3.98E-11
CD5	-0.276	-6.003	2.60E-09	[-0.367, -0.186]	-0.332	-6.911	8.08E-12	[-0.427, -0.238]	1002.979	25.659	0.000	4.35E-11
SCF	-0.302	-5.945	3.67E-09	[-0.402, -0.202]	-0.346	-7.479	2.51E-11	[-0.436, -0.255]	1230.348	28.390	0.000	2.25E-10
CSF-1	0.149	5.816	7.79E-09	[0.099, 0.199]	0.172	6.498	1.36E-10	[0.120, 0.224]	-344.127	23.083	0.000	4.96E-10
VEGFA	0.156	4.598	4.74E-06	[0.089, 0.222]	0.225	6.389	2.51E-10	[0.156, 0.294]	296.896	20.513	0.000	5.37E-09
TNFRSF9	-0.295	-6.039	2.09E-09	[-0.391, -0.199]	-0.269	-5.438	8.55E-08	[-0.366, -0.172]	1135.637	19.959	0.000	1.09E-08
CXCL5	-0.564	-2.931	3.44E-03	[-0.942, -0.187]	-1.183	-5.872	5.65E-09	[-1.577, -0.788]	4286.667	18.083	0.000	5.12E-08
EN-RAGE	-0.399	-4.633	4.01E-06	[-0.568, -0.230]	-0.509	-5.661	1.92E-08	[-0.685, -0.333]	2440.568	16.700	0.000	1.89E-07
CXCL6	-0.479	-4.457	9.12E-06	[-0.689, -0.268]	-0.625	-5.581	3.01E-08	[-0.845, -0.406]	2946.305	16.073	0.000	3.35E-07
CX3CL1	-0.270	-4.888	1.16E-06	[-0.378, -0.162]	-0.298	-5.338	1.45E-07	[-0.407, -0.188]	1413.920	15.780	0.000	4.97E-07

TRAIL	-0.209	-5.343	1.10E-07	[-0.285, -0.132]	-0.192	-4.841	1.69E-06	[-0.270, -0.114]	621.619	15.698	0.000	5.01E-07
CD40	-0.300	-5.330	1.18E-07	[-0.410, -0.189]	-0.266	-4.534	6.40E-06	[-0.382, -0.151]	1461.347	15.087	0.000	7.85E-07
CD244	-0.230	-5.021	5.96E-07	[-0.320, -0.140]	-0.228	-4.895	1.27E-06	[-0.320, -0.137]	988.672	14.721	0.000	1.18E-06
STAMBP	-0.645	-5.213	2.20E-07	[-0.888, -0.403]	-0.510	-3.956	8.12E-05	[-0.763, -0.257]	3270.799	13.803	0.000	2.58E-06
IL7	-0.294	-4.195	2.94E-05	[-0.431, -0.157]	-0.367	-5.008	6.38E-07	[-0.510, -0.223]	1966.895	13.212	0.000	4.45E-06
CD6	-0.399	-4.937	9.11E-07	[-0.558, -0.241]	-0.332	-3.912	9.70E-05	[-0.499, -0.166]	2302.094	12.549	0.000	8.25E-06
IL18	0.231	3.126	1.81E-03	[0.086, 0.377]	0.370	4.974	9.99E-07	[0.224, 0.515]	2088.916	12.418	0.000	1.05E-05
IL-15RA	0.046	1.229	2.19E-01	[-0.028, 0.121]	0.160	4.160	3.70E-05	[0.084, 0.235]	547.796	10.902	0.000	4.15E-05
SIRT2	-0.712	-4.504	7.36E-06	[-1.022, -0.402]	-0.636	-3.849	1.25E-04	[-0.960, -0.312]	3834.757	10.797	0.000	4.22E-05
HGF	-0.016	-0.301	7.64E-01	[-0.122, 0.090]	0.169	3.050	2.39E-03	[0.060, 0.277]	1372.932	9.652	0.000	1.31E-04
NT-3	-0.242	-3.808	1.48E-04	[-0.367, -0.117]	-0.260	-4.363	2.82E-05	[-0.376, -0.143]	1742.303	10.087	0.000	1.35E-04
MCP-1	-0.207	-3.306	9.74E-04	[-0.330, -0.084]	-0.259	-4.495	1.97E-05	[-0.371, -0.146]	1713.553	10.122	0.000	1.51E-04
MMP-10	-0.042	-0.555	5.79E-01	[-0.188, 0.105]	-0.197	-3.166	1.59E-03	[-0.319, -0.075]	2126.917	8.749	0.000	2.85E-04
AXIN1	-0.614	-4.090	4.62E-05	[-0.909, -0.320]	-0.508	-3.222	1.31E-03	[-0.817, -0.199]	3720.048	8.593	0.000	3.25E-04
IFN-γ	-0.283	-2.058	3.98E-02	[-0.553, -0.014]	-0.515	-4.050	1.13E-04	[-0.765, -0.266]	3514.270	8.806	0.000	4.54E-04
CCL19	-0.353	-3.833	1.33E-04	[-0.533, -0.172]	-0.323	-3.561	4.50E-04	[-0.501, -0.145]	2590.156	8.190	0.000	5.26E-04
IL-17C	0.161	1.886	5.96E-02	[-0.006, 0.328]	0.338	3.845	1.29E-04	[0.166, 0.510]	2411.060	7.812	0.000	6.59E-04
IL8	-0.231	-2.655	8.05E-03	[-0.402, -0.060]	-0.349	-3.876	1.14E-04	[-0.525, -0.172]	2461.684	7.516	0.001	8.62E-04
TNFB	-0.176	-3.302	9.89E-04	[-0.281, -0.072]	-0.183	-3.592	4.34E-04	[-0.284, -0.083]	1338.017	7.101	0.001	1.49E-03

CASP-8	-0.384	-3.471	5.39E-04	[-0.601, -0.167]	-0.378	-3.266	1.12E-03	[-0.605, -0.151]	3015.489	6.850	0.001	1.59E-03
ADA	-0.236	-3.551	3.99E-04	[-0.367, -0.106]	-0.183	-2.642	8.36E-03	[-0.319, -0.047]	1847.917	6.375	0.002	2.49E-03
4E-BP1	-0.420	-3.464	5.52E-04	[-0.657, -0.182]	-0.364	-2.987	3.00E-03	[-0.602, -0.125]	3220.209	6.385	0.002	2.50E-03
CXCL9	-0.282	-2.814	4.98E-03	[-0.479, -0.086]	-0.293	-3.119	2.52E-03	[-0.478, -0.109]	2788.670	5.261	0.007	8.87E-03
CCL25	-0.072	-1.018	3.09E-01	[-0.211, 0.067]	0.067	1.063	2.92E-01	[-0.056, 0.190]	1998.789	3.844	0.026	3.43E-02
FGF-21	-0.039	-0.163	8.70E-01	[-0.513, 0.434]	0.446	1.830	6.79E-02	[-0.032, 0.924]	4802.446	3.462	0.032	4.16E-02
ST1A1	-0.385	-2.545	1.10E-02	[-0.681, -0.088]	-0.251	-1.584	1.14E-01	[-0.561, 0.060]	3732.288	3.250	0.039	5.00E-02
IL-18R1	0.021	0.369	7.12E-01	[-0.092, 0.135]	0.111	1.910	5.70E-02	[-0.003, 0.226]	1529.938	2.599	0.075	9.45E-02
Flt3L	0.083	1.633	1.03E-01	[-0.017, 0.183]	0.111	2.265	2.48E-02	[0.015, 0.208]	1232.909	2.572	0.079	9.67E-02
CST5	-0.130	-1.940	5.26E-02	[-0.260, 0.001]	-0.047	-0.719	4.73E-01	[-0.175, 0.081]	1854.042	2.229	0.110	1.33E-01
CXCL1	-0.084	-0.689	4.91E-01	[-0.321, 0.154]	-0.228	-1.795	7.29E-02	[-0.477, 0.021]	3225.681	1.838	0.160	1.90E-01
CXCL11	-0.208	-1.704	8.86E-02	[-0.447, 0.031]	-0.189	-1.490	1.36E-01	[-0.438, 0.060]	3236.246	1.566	0.209	2.45E-01
CCL3	0.077	0.866	3.86E-01	[-0.097, 0.251]	0.145	1.613	1.07E-01	[-0.031, 0.322]	2506.880	1.345	0.261	3.00E-01
CCL4	-0.102	-1.409	1.59E-01	[-0.244, 0.040]	-0.095	-1.278	2.02E-01	[-0.240, 0.051]	2040.403	1.094	0.335	3.79E-01
CCL20	-0.165	-1.336	1.82E-01	[-0.408, 0.077]	-0.087	-0.839	4.05E-01	[-0.289, 0.116]	3278.035	0.921	0.406	4.40E-01
IL10	0.019	0.229	8.19E-01	[-0.145, 0.184]	-0.064	-0.797	4.28E-01	[-0.220, 0.093]	2380.127	0.903	0.408	4.40E-01
MMP-1	0.037	0.268	7.89E-01	[-0.233, 0.307]	0.167	1.161	2.46E-01	[-0.115, 0.449]	3519.963	0.903	0.406	4.40E-01
FGF-19	0.073	0.565	5.72E-01	[-0.181, 0.327]	-0.043	-0.356	7.22E-01	[-0.283, 0.196]	3378.565	0.703	0.497	5.23E-01
OSM	0.136	1.164	2.45E-01	[-0.093, 0.366]	0.084	0.695	4.88E-01	[-0.153, 0.320]	3141.095	0.683	0.505	5.23E-01

TNF	-0.063	-0.958	3.38E-01	[-0.191, 0.066]	-0.011	-0.162	8.71E-01	[-0.138, 0.117]	1809.609	0.679	0.508	5.23E-01
IL6	-0.050	-0.519	6.04E-01	[-0.241, 0.140]	-0.072	-0.721	4.71E-01	[-0.268, 0.124]	2713.506	0.261	0.770	7.82E-01
TGF-α	0.006	0.120	9.04E-01	[-0.092, 0.104]	-0.002	-0.035	9.72E-01	[-0.104, 0.101]	1194.707	0.019	0.981	9.81E-01

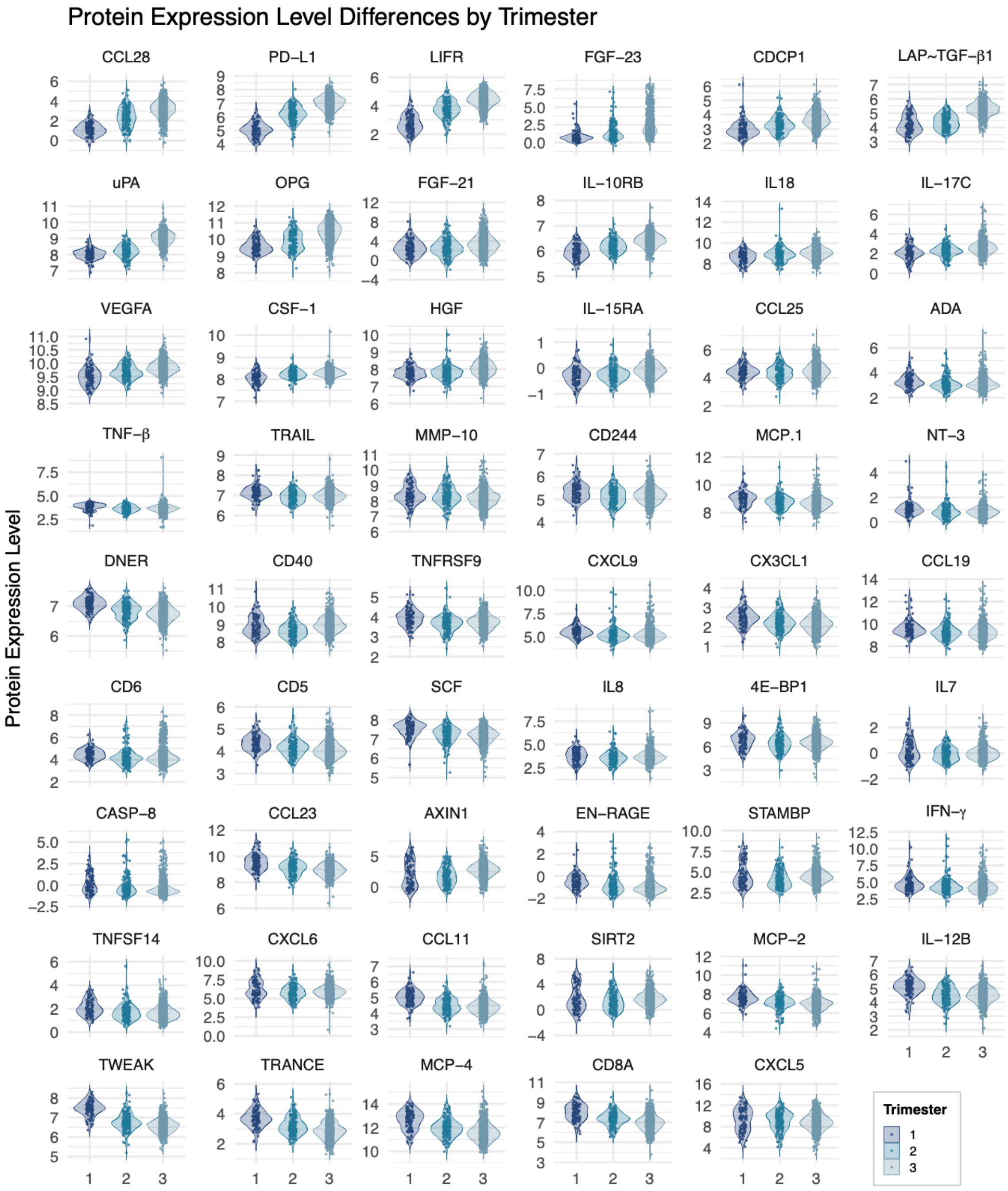

Supplementary Figure 3. Trimester-related differences in protein expression levels, sorted by significance. Univariate mixed-effects regression models detected 53 inflammatory proteins that showed significant differences across trimesters at 5% FDR. Models included maternal age at blood draw, gravidity, and infant sex as covariates, with site modeled as a random intercept to account for site-level heterogeneity. Protein expression levels are displayed by trimester (first, second, and third), with violin plots illustrating the distribution of values and overlaid points representing individual samples. Violin plots illustrate clear shifts in the distribution of protein expression levels across the first, second, and third trimesters.

Supplementary Table 4. Main effect of maternal age on expression level for each protein. Abbreviations: AIC, Akaike information criterion; CI, confidence interval; FDR, false discovery rate.

Protein	B estimate	t statistic	95% CI	AIC	p (uncorrected)	p (FDR-adjusted)
Flt3L	0.012	4.653	[0.007, 0.017]	1232.280	0.000	<0.001
FGF-23	-0.045	-4.494	[-0.065, -0.025]	4327.788	0.000	<0.001
IL-10RB	0.005	3.063	[0.002, 0.009]	281.164	0.002	0.039
PD-L1	0.010	3.080	[0.004, 0.017]	1752.450	0.002	0.039
CCL20	-0.017	-2.882	[-0.029, -0.006]	3281.326	0.004	0.050
FGF-21	0.035	2.810	[0.011, 0.059]	4795.426	0.005	0.050
MCP-4	0.014	2.827	[0.004, 0.024]	2689.662	0.005	0.050
FGF-19	0.018	2.729	[0.005, 0.031]	3380.169	0.007	0.057
LIF-R	0.009	2.600	[0.002, 0.015]	1808.548	0.010	0.071
MCP-1	0.008	2.579	[0.002, 0.014]	1725.321	0.010	0.071
CD40	0.007	2.369	[0.001, 0.013]	1475.780	0.018	0.083
CDCP1	0.009	2.427	[0.002, 0.015]	2017.990	0.016	0.083
CXCL9	0.012	2.433	[0.002, 0.022]	2796.562	0.015	0.083
HGF	0.007	2.397	[0.001, 0.012]	1357.235	0.017	0.083
SCF	0.006	2.399	[0.001, 0.011]	1239.109	0.017	0.083
VEGFA	0.004	2.296	[0.001, 0.008]	286.763	0.022	0.094
CCL11	0.007	2.214	[0.001, 0.013]	1652.152	0.027	0.110
CCL25	0.008	2.180	[0.001, 0.014]	1997.570	0.030	0.114
TRAIL	-0.004	-2.156	[-0.008, 0.000]	637.669	0.031	0.114
MMP-1	0.015	2.043	[0.001, 0.029]	3522.076	0.041	0.142
EN-RAGE	-0.009	-1.932	[-0.018, 0.000]	2433.329	0.054	0.176
CST5	0.007	1.913	[0.000, 0.013]	1858.724	0.056	0.176
MMP-10	0.007	1.881	[0.000, 0.014]	2127.907	0.060	0.177
TNFSF14	0.007	1.871	[0.000, 0.015]	2122.743	0.062	0.177
CD5	-0.004	-1.779	[-0.009, 0.000]	1000.560	0.076	0.201
uPA	0.004	1.784	[0.000, 0.009]	947.791	0.075	0.201
TRANCE	-0.006	-1.725	[-0.013, 0.001]	1963.576	0.085	0.217
CD8A	-0.008	-1.612	[-0.018, 0.002]	2682.904	0.107	0.264
CSF-1	0.002	1.526	[-0.001, 0.005]	-341.897	0.127	0.274
CXCL6	0.009	1.565	[-0.002, 0.020]	2957.322	0.118	0.274
DNER	0.003	1.554	[-0.001, 0.006]	318.439	0.121	0.274
IL-18R1	-0.005	-1.535	[-0.010, 0.001]	1525.699	0.125	0.274
CASP-8	0.009	1.457	[-0.003, 0.020]	3019.536	0.145	0.295
IL-17C	-0.007	-1.469	[-0.015, 0.002]	2398.564	0.142	0.295
IL7	0.005	1.424	[-0.002, 0.013]	1972.134	0.155	0.298
MCP-2	0.007	1.422	[-0.003, 0.017]	2650.408	0.155	0.298
IL-15RA	0.003	1.406	[-0.001, 0.007]	535.796	0.160	0.298

OSM	0.009	1.390	[-0.004, 0.021]	3143.508	0.165	0.299
IL8	0.006	1.288	[-0.003, 0.015]	2473.203	0.198	0.350
AXIN1	0.010	1.219	[-0.006, 0.025]	3720.330	0.223	0.385
IL6	-0.006	-1.169	[-0.016, 0.004]	2713.414	0.243	0.399
NT-3	-0.004	-1.178	[-0.010, 0.003]	1755.254	0.240	0.399
CCL23	0.004	1.120	[-0.003, 0.010]	1755.134	0.263	0.421
CCL3	0.005	1.093	[-0.004, 0.014]	2506.297	0.275	0.421
CCL4	0.004	1.101	[-0.003, 0.012]	2042.125	0.271	0.421
CD244	0.002	0.959	[-0.002, 0.007]	981.910	0.338	0.486
CX3CL1	0.003	0.962	[-0.003, 0.008]	1427.123	0.336	0.486
IL10	-0.004	-0.965	[-0.012, 0.004]	2381.891	0.336	0.486
CCL28	-0.005	-0.870	[-0.016, 0.006]	3076.633	0.385	0.532
IL18	-0.003	-0.880	[-0.011, 0.004]	2094.983	0.379	0.532
TNFB	-0.002	-0.851	[-0.008, 0.003]	1345.834	0.395	0.535
TWEAK	0.002	0.748	[-0.003, 0.007]	1024.157	0.455	0.604
TNFRSF9	-0.002	-0.727	[-0.007, 0.003]	1165.991	0.467	0.608
CXCL11	-0.003	-0.482	[-0.016, 0.010]	3240.201	0.630	0.733
IL-12B	0.002	0.535	[-0.006, 0.010]	2141.480	0.593	0.733
LAP-TGF-β-1	0.002	0.489	[-0.005, 0.008]	1717.636	0.625	0.733
ST1A1	0.004	0.472	[-0.012, 0.019]	3732.799	0.637	0.733
STAMBP	0.004	0.557	[-0.009, 0.016]	3277.915	0.577	0.733
TGF-α	-0.001	-0.483	[-0.006, 0.004]	1193.492	0.629	0.733
TNF	0.002	0.530	[-0.005, 0.008]	1811.004	0.596	0.733
SIRT2	0.003	0.369	[-0.013, 0.019]	3835.821	0.712	0.806
ADA	0.001	0.251	[-0.006, 0.008]	1856.512	0.802	0.865
CD6	-0.001	-0.225	[-0.009, 0.007]	2311.437	0.822	0.865
CXCL5	0.002	0.219	[-0.018, 0.022]	4274.840	0.827	0.865
OPG	0.001	0.279	[-0.006, 0.008]	2021.292	0.781	0.865
4E-BP1	-0.002	-0.244	[-0.014, 0.011]	3226.610	0.807	0.865
IFN-γ	0.001	0.183	[-0.012, 0.015]	3521.805	0.855	0.881
CXCL1	0.001	0.158	[-0.012, 0.014]	3222.126	0.874	0.887
CCL19	0.000	0.026	[-0.009, 0.009]	2600.078	0.979	0.979

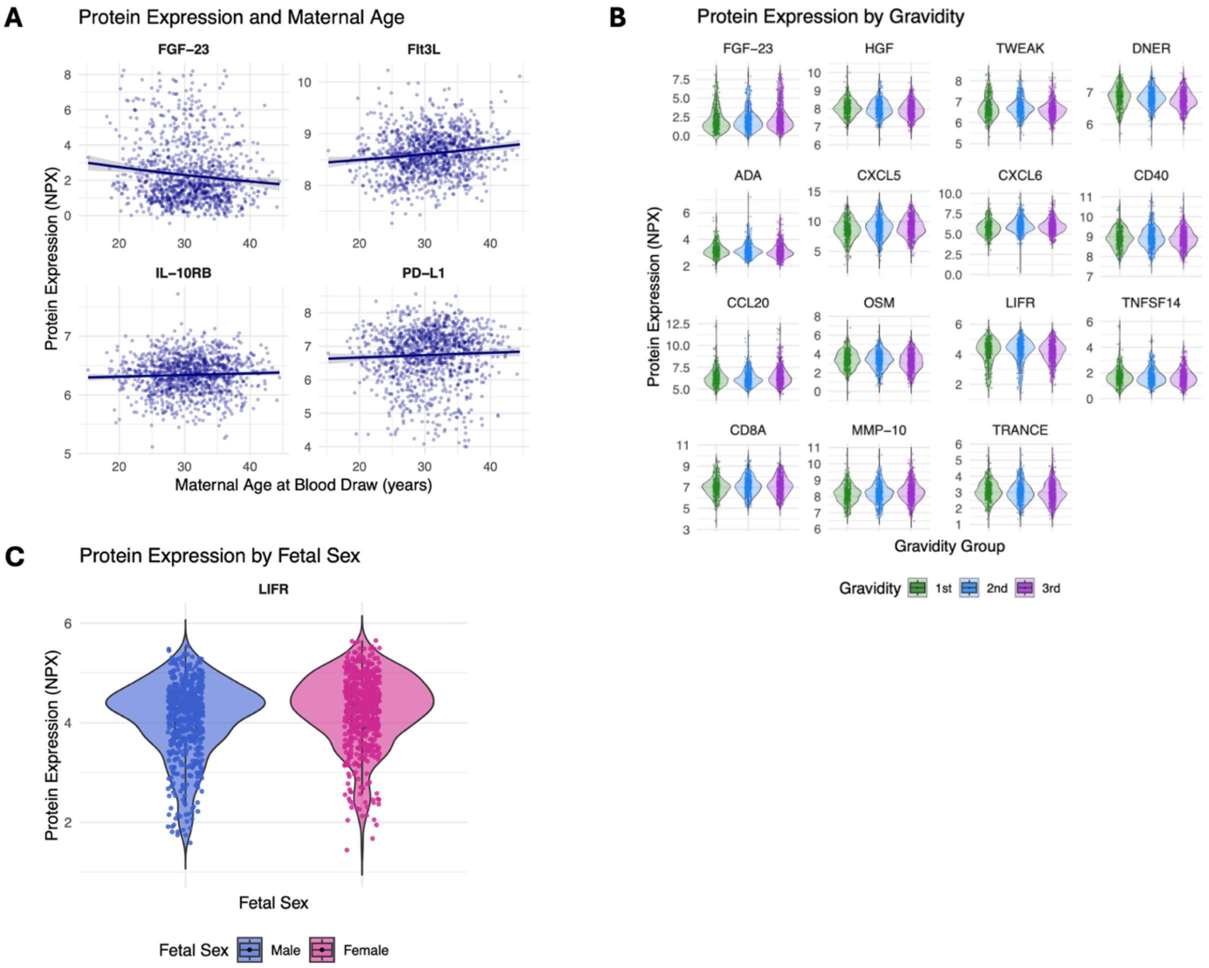

Supplementary Figure 4. Associations of fetal sex, gravidity, and maternal age with overall protein expression levels. Scatterplots (bottom left) show linear associations between maternal age at blood draw and protein expression (A). Shaded bands represent 95% confidence intervals. Violin plots display protein expression (Normalized Protein eXpression, NPX) stratified by gravidity group (B) and fetal sex (C). Linear mixed-effects models tested main effects of fetal sex, gravidity, and maternal age on overall protein levels, adjusting for the other covariates as appropriate and including a random intercept for ECHO site. Only proteins with significant associations ($p<0.05$) are shown. Full model results are provided below. Four proteins showed significant associations with maternal age at blood draw: FGF-23, Flt3L, IL-10RB, and PD-L1. FGF-23 expression decreased with maternal age, while Flt3L, IL-10RB, and PD-L1 expression increased with maternal age (A). We also identified 15 proteins that differed by gravidity status: FGF-23, HGF, TWEAK, DNER, ADA, CXCL5, CXCL6, CD40, CCL20, OSM, LIFR, TNFSF14, CD8A, MMP-10, and TRANCE (B). In contrast, we identified one protein that differed by fetal sex: LIFR expression was higher in pregnancies with female fetuses (C). See Supplementary Tables 4–6 for full results.

Supplementary Table 5. Main effect of gravidity on expression level for each protein. Abbreviations: AIC, Akaike information criterion; CI, confidence interval; FDR, false discovery rate.

Protein	G1–G2 β estimate	G1–G2 t statistic	G1–G2 p	G1–G2 95% CI	G1–G3 Β estimate	G1–G3 t statistic	G1–G3 p	G1–G3 95% CI	AIC	Omnibus F	Omnibus p (uncorrected)	Omnibus p (FDR-adjusted)
HGF	-0.066	-1.799	0.072	[-0.138, 0.006]	-0.148	-4.131	0.000	[-0.219, -0.078]	1357.235	9.107	<0.001	0.003
FGF-23	-0.021	-0.156	0.876	[-0.283, 0.241]	0.416	3.219	0.001	[0.163, 0.669]	4327.788	9.285	<0.001	0.003
TWEAK	0.021	0.655	0.513	[-0.042, 0.083]	-0.089	-2.858	0.004	[-0.150, -0.028]	1024.157	9.200	<0.001	0.003
DNER	-0.020	-0.874	0.382	[-0.066, 0.025]	-0.080	-3.526	0.000	[-0.124, -0.035]	318.439	7.796	<0.001	0.008
ADA	0.018	0.391	0.696	[-0.072, 0.108]	-0.120	-2.670	0.008	[-0.209, -0.032]	1856.512	7.198	<0.001	0.011
CD40	-0.007	-0.177	0.860	[-0.083, 0.069]	-0.106	-2.775	0.006	[-0.181, -0.031]	1475.780	5.942	0.003	0.026
CCL20	0.011	0.142	0.887	[-0.145, 0.168]	0.206	2.683	0.007	[0.055, 0.356]	3281.326	5.725	0.003	0.026
CXCL6	0.186	2.505	0.012	[0.040, 0.331]	-0.012	-0.168	0.867	[-0.155, 0.131]	2957.322	5.718	0.003	0.026
CXCL5	0.339	2.577	0.010	[0.081, 0.597]	-0.023	-0.181	0.856	[-0.277, 0.230]	4274.840	6.076	0.002	0.026
OSM	-0.063	-0.781	0.435	[-0.220, 0.095]	-0.237	-3.004	0.003	[-0.391, -0.082]	3143.508	5.573	0.004	0.027
LIF-R	-0.022	-0.489	0.625	[-0.110, 0.066]	-0.122	-2.813	0.005	[-0.207, -0.037]	1808.548	5.378	0.005	0.030
TNFSF14	0.014	0.273	0.785	[-0.087, 0.115]	-0.114	-2.249	0.025	[-0.213, -0.015]	2122.743	4.942	0.007	0.042
MMP-10	0.053	1.101	0.271	[-0.042, 0.148]	0.136	2.926	0.004	[0.045, 0.227]	2127.907	4.793	0.008	0.043
CD8A	0.168	2.561	0.011	[0.040, 0.297]	0.191	2.958	0.003	[0.064, 0.318]	2682.904	4.767	0.009	0.043
TRANCE	-0.074	-1.546	0.122	[-0.167, 0.020]	-0.139	-3.000	0.003	[-0.230, -0.048]	1963.576	4.610	0.010	0.047
CX3CL1	-0.050	-1.322	0.186	[-0.125, 0.024]	-0.106	-2.861	0.004	[-0.178, -0.033]	1427.123	4.298	0.014	0.060

CD244	0.062	1.990	0.047	[0.001, 0.124]	-0.011	-0.364	0.716	[-0.072, 0.049]	981.910	4.194	0.015	0.062
IL7	0.092	1.913	0.056	[-0.002, 0.187]	-0.021	-0.440	0.660	[-0.114, 0.072]	1972.134	4.116	0.017	0.063
TNFRSF9	0.011	0.333	0.739	[-0.055, 0.078]	-0.063	-1.901	0.058	[-0.128, 0.002]	1165.991	3.798	0.023	0.083
CASP-8	0.104	1.360	0.174	[-0.046, 0.253]	-0.070	-0.934	0.351	[-0.217, 0.077]	3019.536	3.622	0.027	0.093
uPA	0.006	0.190	0.849	[-0.055, 0.066]	-0.058	-1.905	0.057	[-0.117, 0.002]	947.791	3.451	0.032	0.105
CXCL1	0.193	2.325	0.020	[0.030, 0.356]	0.047	0.575	0.565	[-0.113, 0.207]	3222.126	3.377	0.034	0.108
CST5	-0.062	-1.369	0.171	[-0.152, 0.027]	-0.109	-2.464	0.014	[-0.195, -0.022]	1858.724	3.068	0.047	0.141
Flt3L	0.068	1.965	0.050	[0.000, 0.136]	0.005	0.138	0.890	[-0.061, 0.070]	1232.280	2.976	0.052	0.148
IL-10RB	-0.002	-0.098	0.922	[-0.048, 0.043]	-0.042	-1.841	0.066	[-0.086, 0.003]	281.164	2.653	0.071	0.188
IL18	0.084	1.648	0.100	[-0.016, 0.183]	0.114	2.306	0.021	[0.017, 0.211]	2094.983	2.687	0.069	0.188
OPG	0.040	0.829	0.408	[-0.055, 0.135]	0.100	2.155	0.032	[0.009, 0.192]	2021.292	2.578	0.077	0.198
MCP-4	-0.058	-0.873	0.383	[-0.187, 0.072]	-0.136	-2.098	0.036	[-0.263, -0.009]	2689.662	2.375	0.093	0.230
SCF	0.002	0.055	0.956	[-0.063, 0.067]	-0.052	-1.619	0.107	[-0.114, 0.011]	1239.109	2.331	0.100	0.238
NT-3	-0.030	-0.701	0.484	[-0.115, 0.055]	-0.083	-2.000	0.046	[-0.165, -0.002]	1755.254	2.279	0.103	0.238
IFN-γ	-0.156	-1.670	0.095	[-0.338, 0.027]	-0.012	-0.137	0.891	[-0.188, 0.163]	3521.805	2.124	0.121	0.269
PD-L1	-0.017	-0.390	0.697	[-0.103, 0.069]	-0.077	-1.788	0.074	[-0.161, 0.007]	1752.450	2.065	0.127	0.275
CCL28	0.019	0.244	0.807	[-0.130, 0.167]	0.121	1.671	0.096	[-0.021, 0.264]	3076.633	1.975	0.141	0.294
LAP-TGF-β-1	-0.037	-0.860	0.390	[-0.122, 0.048]	-0.079	-1.857	0.064	[-0.162, 0.004]	1717.636	1.808	0.164	0.334
TGF-α	-0.050	-1.467	0.143	[-0.118, 0.017]	-0.059	-1.744	0.081	[-0.125, 0.007]	1193.492	1.630	0.196	0.387

TRAIL	0.037	1.388	0.166	[-0.015, 0.090]	0.000	0.011	0.991	[-0.051, 0.052]	637.669	1.597	0.203	0.389
TNF	0.018	0.409	0.682	[-0.070, 0.106]	0.068	1.567	0.118	[-0.017, 0.154]	1811.004	1.516	0.220	0.410
IL8	0.100	1.667	0.096	[-0.018, 0.218]	0.066	1.129	0.259	[-0.049, 0.182]	2473.203	1.394	0.249	0.451
CCL4	-0.064	-1.296	0.195	[-0.162, 0.033]	-0.076	-1.567	0.117	[-0.172, 0.019]	2042.125	1.304	0.272	0.469
CCL3	-0.049	-0.809	0.419	[-0.168, 0.070]	0.034	0.575	0.565	[-0.082, 0.151]	2506.297	1.321	0.267	0.469
IL6	0.003	0.049	0.961	[-0.127, 0.134]	0.080	1.234	0.218	[-0.047, 0.208]	2713.414	1.212	0.298	0.501
STAMBP	0.068	0.795	0.427	[-0.099, 0.235]	-0.039	-0.462	0.644	[-0.203, 0.126]	3277.915	1.097	0.334	0.549
SIRT2	0.052	0.474	0.635	[-0.161, 0.264]	-0.077	-0.719	0.472	[-0.286, 0.133]	3835.821	0.981	0.375	0.602
IL-12B	-0.040	-0.778	0.437	[-0.142, 0.061]	-0.067	-1.320	0.187	[-0.167, 0.033]	2141.480	0.874	0.417	0.643
FGF-19	-0.031	-0.358	0.721	[-0.203, 0.141]	-0.098	-1.165	0.244	[-0.264, 0.067]	3380.169	0.806	0.447	0.643
CCL19	-0.024	-0.380	0.704	[-0.148, 0.100]	-0.072	-1.178	0.239	[-0.192, 0.048]	2600.078	0.809	0.446	0.643
CSF-1	0.018	1.037	0.300	[-0.016, 0.053]	0.001	0.037	0.970	[-0.033, 0.034]	-341.897	0.861	0.423	0.643
AXIN1	0.054	0.525	0.600	[-0.148, 0.256]	-0.059	-0.577	0.564	[-0.258, 0.140]	3720.330	0.830	0.436	0.643
CCL25	-0.051	-1.103	0.272	[-0.142, 0.040]	-0.044	-0.996	0.320	[-0.131, 0.043]	1997.570	0.679	0.508	0.662
MCP-1	-0.023	-0.536	0.592	[-0.105, 0.060]	-0.047	-1.174	0.241	[-0.127, 0.032]	1725.321	0.729	0.483	0.662
CD5	0.037	1.176	0.240	[-0.025, 0.099]	0.031	0.990	0.323	[-0.030, 0.092]	1000.560	0.741	0.477	0.662
IL-18R1	0.044	1.116	0.265	[-0.033, 0.122]	0.037	0.967	0.334	[-0.038, 0.113]	1525.699	0.678	0.508	0.662
MMP-1	0.093	0.986	0.324	[-0.092, 0.279]	0.012	0.128	0.898	[-0.171, 0.195]	3522.076	0.693	0.500	0.662
IL-15RA	-0.010	-0.400	0.689	[-0.061, 0.040]	0.014	0.569	0.569	[-0.035, 0.064]	535.796	0.648	0.523	0.663

CD6	0.060	1.074	0.283	[-0.049, 0.169]	0.023	0.411	0.681	[-0.085, 0.130]	2311.437	0.638	0.529	0.663
ST1A1	0.105	1.016	0.310	[-0.098, 0.309]	0.036	0.354	0.723	[-0.164, 0.236]	3732.799	0.586	0.556	0.686
FGF-21	-0.016	-0.097	0.923	[-0.338, 0.306]	0.113	0.707	0.480	[-0.200, 0.426]	4795.426	0.501	0.606	0.733
VEGFA	-0.001	-0.022	0.982	[-0.046, 0.045]	-0.017	-0.735	0.463	[-0.061, 0.028]	286.763	0.435	0.648	0.733
TNFB	0.009	0.246	0.806	[-0.062, 0.080]	-0.019	-0.545	0.586	[-0.088, 0.050]	1345.834	0.446	0.641	0.733
CXCL9	0.018	0.266	0.791	[-0.115, 0.152]	0.058	0.887	0.376	[-0.070, 0.187]	2796.562	0.470	0.625	0.733
CXCL11	0.077	0.920	0.358	[-0.087, 0.241]	0.039	0.469	0.639	[-0.123, 0.200]	3240.201	0.434	0.648	0.733
CCL23	0.026	0.585	0.559	[-0.060, 0.112]	-0.004	-0.098	0.922	[-0.089, 0.080]	1755.134	0.355	0.701	0.780
MCP-2	-0.050	-0.776	0.438	[-0.177, 0.077]	-0.044	-0.699	0.485	[-0.168, 0.080]	2650.408	0.336	0.714	0.782
IL-17C	-0.038	-0.658	0.510	[-0.152, 0.076]	-0.036	-0.635	0.525	[-0.148, 0.075]	2398.564	0.256	0.774	0.826
CCL11	-0.030	-0.706	0.480	[-0.111, 0.052]	-0.017	-0.407	0.684	[-0.096, 0.063]	1652.152	0.251	0.778	0.826
4E-BP1	0.025	0.296	0.768	[-0.139, 0.188]	-0.023	-0.287	0.774	[-0.183, 0.136]	3226.610	0.233	0.792	0.828
CDCP1	0.006	0.134	0.894	[-0.086, 0.099]	-0.016	-0.363	0.717	[-0.105, 0.072]	2017.990	0.180	0.835	0.860
IL10	-0.023	-0.409	0.683	[-0.132, 0.086]	-0.028	-0.528	0.598	[-0.132, 0.076]	2381.891	0.143	0.866	0.879
EN-RAGE	0.018	0.310	0.756	[-0.097, 0.134]	0.000	0.004	0.997	[-0.113, 0.114]	2433.329	0.079	0.924	0.924

Supplementary Table 6. Main effect of fetal sex on expression level for each protein. Abbreviations: AIC, Akaike information criterion; CI, confidence interval; FDR, false discovery rate.

Protein	B estimate	t statistic	95% CI	AIC	p (uncorrected)	p (FDR-adjusted)
LIF-R	0.135	4.415	[0.075, 0.194]	1808.548	0.000	0.001
CD244	-0.061	-2.884	[-0.103, -0.020]	981.910	0.004	0.081
IL-10RB	-0.047	-3.030	[-0.078, -0.017]	281.164	0.002	0.081
IL10	-0.111	-2.834	[-0.188, -0.034]	2381.891	0.005	0.081
IL-18R1	-0.066	-2.444	[-0.119, -0.013]	1525.699	0.015	0.191
TNFRSF9	-0.055	-2.399	[-0.100, -0.010]	1165.991	0.017	0.191
PD-L1	0.069	2.336	[0.011, 0.128]	1752.450	0.020	0.194
CD40	-0.060	-2.284	[-0.111, -0.009]	1475.780	0.023	0.194
IL-15RA	-0.038	-2.158	[-0.072, -0.003]	535.796	0.031	0.239
CD6	-0.070	-1.850	[-0.144, 0.004]	2311.437	0.065	0.446
CCL25	-0.055	-1.664	[-0.120, 0.010]	1997.570	0.096	0.514
CXCL9	-0.078	-1.662	[-0.170, 0.014]	2796.562	0.097	0.514
TRANCE	-0.056	-1.728	[-0.120, 0.008]	1963.576	0.084	0.514
CD5	-0.033	-1.544	[-0.075, 0.009]	1000.560	0.123	0.529
FGF-23	0.142	1.549	[-0.038, 0.321]	4327.788	0.122	0.529
IFN-γ	-0.102	-1.585	[-0.228, 0.024]	3521.805	0.113	0.529
LAP-TGF-β-1	-0.043	-1.476	[-0.100, 0.014]	1717.636	0.140	0.569
CASP-8	0.072	1.404	[-0.029, 0.173]	3019.536	0.161	0.589
MMP-10	-0.049	-1.399	[-0.118, 0.020]	2127.907	0.162	0.589
CXCL5	0.116	1.301	[-0.058, 0.290]	4274.840	0.193	0.635
IL18	-0.045	-1.311	[-0.113, 0.022]	2094.983	0.190	0.635
CXCL1	0.064	1.139	[-0.046, 0.174]	3222.126	0.255	0.705
IL-12B	-0.042	-1.183	[-0.111, 0.027]	2141.480	0.237	0.705
IL-17C	0.045	1.138	[-0.032, 0.122]	2398.564	0.255	0.705
uPA	-0.025	-1.210	[-0.066, 0.016]	947.791	0.227	0.705
HGF	-0.028	-1.100	[-0.077, 0.022]	1357.235	0.271	0.719
OSM	0.059	1.078	[-0.048, 0.165]	3143.508	0.281	0.719
MCP-2	-0.045	-1.035	[-0.132, 0.041]	2650.408	0.301	0.742
AXIN1	-0.069	-0.989	[-0.205, 0.068]	3720.330	0.323	0.766
FGF-19	0.059	0.969	[-0.060, 0.177]	3380.169	0.333	0.766
CCL23	-0.028	-0.932	[-0.086, 0.031]	1755.134	0.351	0.782
CCL19	-0.032	-0.747	[-0.117, 0.052]	2600.078	0.455	0.873
CD8A	0.036	0.820	[-0.051, 0.124]	2682.904	0.412	0.873
CXCL11	-0.043	-0.766	[-0.154, 0.068]	3240.201	0.444	0.873
EN-RAGE	0.031	0.778	[-0.047, 0.109]	2433.329	0.437	0.873
TWEAK	-0.017	-0.768	[-0.059, 0.026]	1024.157	0.443	0.873

DNER	-0.011	-0.685	[-0.042, 0.020]	318.439	0.493	0.901
FGF-21	-0.074	-0.664	[-0.294, 0.145]	4795.426	0.507	0.901
IL8	0.027	0.660	[-0.053, 0.106]	2473.203	0.509	0.901
CDCP1	-0.020	-0.591	[-0.085, 0.046]	2017.990	0.555	0.934
ST1A1	0.043	0.608	[-0.095, 0.180]	3732.799	0.543	0.934
IL6	0.025	0.546	[-0.064, 0.113]	2713.414	0.585	0.938
NT-3	-0.015	-0.508	[-0.074, 0.043]	1755.254	0.611	0.938
TNF	-0.015	-0.508	[-0.075, 0.044]	1811.004	0.612	0.938
TNFB	-0.014	-0.543	[-0.062, 0.035]	1345.834	0.587	0.938
TRAIL	-0.009	-0.486	[-0.045, 0.027]	637.669	0.627	0.941
ADA	0.012	0.380	[-0.049, 0.073]	1856.512	0.704	0.948
CCL20	-0.013	-0.218	[-0.126, 0.101]	3281.326	0.827	0.948
CCL28	-0.010	-0.187	[-0.114, 0.094]	3076.633	0.852	0.948
CCL4	0.006	0.169	[-0.060, 0.072]	2042.125	0.866	0.948
CSF-1	0.005	0.438	[-0.018, 0.029]	-341.897	0.662	0.948
CST5	-0.010	-0.335	[-0.072, 0.051]	1858.724	0.737	0.948
CX3CL1	-0.008	-0.319	[-0.059, 0.042]	1427.123	0.749	0.948
CXCL6	-0.010	-0.204	[-0.108, 0.088]	2957.322	0.839	0.948
Flt3L	0.008	0.354	[-0.038, 0.055]	1232.280	0.723	0.948
IL7	-0.015	-0.458	[-0.079, 0.049]	1972.134	0.647	0.948
MCP-1	0.006	0.219	[-0.051, 0.064]	1725.321	0.826	0.948
MMP-1	0.018	0.275	[-0.108, 0.143]	3522.076	0.783	0.948
SCF	-0.004	-0.179	[-0.051, 0.042]	1239.109	0.858	0.948
SIRT2	-0.016	-0.212	[-0.159, 0.128]	3835.821	0.832	0.948
STAMBP	-0.018	-0.310	[-0.131, 0.095]	3277.915	0.756	0.948
TGF-α	0.005	0.234	[-0.040, 0.051]	1193.492	0.815	0.948
VEGFA	-0.007	-0.417	[-0.037, 0.024]	286.763	0.677	0.948
CCL11	0.003	0.103	[-0.053, 0.059]	1652.152	0.918	0.951
CCL3	0.005	0.111	[-0.076, 0.085]	2506.297	0.912	0.951
MCP-4	0.005	0.110	[-0.082, 0.092]	2689.662	0.912	0.951
OPG	-0.003	-0.097	[-0.069, 0.062]	2021.292	0.923	0.951
4E-BP1	0.003	0.053	[-0.108, 0.114]	3226.610	0.958	0.972
TNFSF14	-0.001	-0.031	[-0.069, 0.067]	2122.743	0.975	0.975

Supplementary Table 7. Linear models for continuous gestational age. Abbreviations: AIC, Akaike information criterion; CI, confidence interval; FDR, false discovery rate.

Protein	B estimate	t statistic	95% CI	p (uncorrected)	p (FDR-adjusted)	Model fit
CCL28	0.074	22.948	[0.067, 0.080]	4.12E-09	1.14E-08	AIC=3042.2; R2m=0.324; R2c=0.324
PD-L1	0.072	27.334	[0.067, 0.077]	5.66E-79	3.90E-77	AIC=1714.1; R2m=0.581; R2c=0.602
FGF-23	0.065	9.829	[0.052, 0.079]	4.97E-06	1.04E-05	AIC=4301.3; R2m=0.143; R2c=0.148
LIF-R	0.065	25.677	[0.060, 0.070]	5.07E-43	1.17E-41	AIC=1769.6; R2m=0.526; R2c=0.536
FGF-21	0.043	4.702	[0.025, 0.061]	1.44E-05	2.83E-05	AIC=4772.1; R2m=0.043; R2c=0.062
uPA	0.039	20.028	[0.035, 0.043]	7.54E-74	2.60E-72	AIC=906.6; R2m=0.419; R2c=0.513
LAP-TGF-β1	0.038	14.079	[0.033, 0.044]	8.76E-41	1.51E-39	AIC=1680.5; R2m=0.261; R2c=0.385
OPG	0.036	17.408	[0.032, 0.040]	8.74E-07	2.15E-06	AIC=1981.7; R2m=0.228; R2c=0.229
CDCP1	0.029	14.236	[0.025, 0.033]	9.75E-12	3.96E-11	AIC=1977.7; R2m=0.155; R2c=0.155
IL-17C	0.020	5.529	[0.013, 0.027]	5.56E-08	1.42E-07	AIC=2364.1; R2m=0.058; R2c=0.129
IL-10RB	0.017	12.111	[0.014, 0.020]	2.29E-28	2.25E-27	AIC=235.4; R2m=0.222; R2c=0.271
HGF	0.014	6.424	[0.010, 0.018]	1.81E-09	5.67E-09	AIC=1316.4; R2m=0.083; R2c=0.114
IL18	0.013	4.536	[0.008, 0.019]	1.26E-05	2.56E-05	AIC=2058.0; R2m=0.043; R2c=0.073
VEGFA	0.011	7.298	[0.008, 0.013]	8.12E-13	4.00E-12	AIC=241.7; R2m=0.093; R2c=0.199
IL-15RA	0.009	5.965	[0.006, 0.012]	1.23E-08	3.26E-08	AIC=490.8; R2m=0.065; R2c=0.101
IL-18R1	0.008	3.589	[0.004, 0.012]	6.44E-04	1.03E-03	AIC=1485.4; R2m=0.029; R2c=0.050
CCL3	0.008	2.136	[0.001, 0.015]	3.43E-02	4.30E-02	AIC=2471.7; R2m=0.012; R2c=0.051
CCL25	0.007	3.757	[0.004, 0.011]	1.81E-04	3.04E-04	AIC=1956.8; R2m=0.018; R2c=0.018
CSF-1	0.007	6.640	[0.005, 0.009]	9.30E-11	3.38E-10	AIC=-390.7; R2m=0.080; R2c=0.150
MMP-1	0.007	1.194	[-0.005, 0.019]	2.33E-01	2.64E-01	AIC=3494.3; R2m=0.008; R2c=0.175
IL6	0.005	1.350	[-0.002, 0.013]	1.78E-01	2.05E-01	AIC=2680.3; R2m=0.008; R2c=0.066
Flt3L	0.005	2.584	[0.001, 0.008]	1.26E-02	1.67E-02	AIC=1189.7; R2m=0.036; R2c=0.047
OSM	0.003	0.539	[-0.007, 0.012]	5.90E-01	6.17E-01	AIC=3113.0; R2m=0.013; R2c=0.092
IL10	0.003	1.129	[-0.002, 0.007]	2.59E-01	2.88E-01	AIC=2343.3; R2m=0.009; R2c=0.009

TGF-α	0.002	0.944	[-0.002, 0.006]	3.45E-01	3.78E-01	AIC=1153.9; R2m=0.005; R2c=0.221
TNF	0.001	0.442	[-0.004, 0.006]	6.59E-01	6.79E-01	AIC=1772.3; R2m=0.004; R2c=0.030
CST5	0.000	-0.087	[-0.005, 0.005]	9.32E-01	9.32E-01	AIC=1819.5; R2m=0.007; R2c=0.019
CCL20	-0.001	-0.401	[-0.008, 0.005]	6.88E-01	6.98E-01	AIC=3247.2; R2m=0.015; R2c=0.015
CCL4	-0.003	-0.877	[-0.009, 0.003]	3.81E-01	4.11E-01	AIC=2005.5; R2m=0.005; R2c=0.062
CXCL11	-0.004	-0.688	[-0.014, 0.007]	4.91E-01	5.22E-01	AIC=3210.8; R2m=0.002; R2c=0.145
TNFB	-0.005	-2.577	[-0.009, -0.001]	1.34E-02	1.75E-02	AIC=1303.8; R2m=0.011; R2c=0.021
NT-3	-0.006	-2.637	[-0.010, -0.001]	1.41E-02	1.80E-02	AIC=1714.9; R2m=0.016; R2c=0.022
TNFRSF9	-0.006	-2.872	[-0.010, -0.002]	4.55E-03	6.54E-03	AIC=1124.4; R2m=0.029; R2c=0.069
CXCL9	-0.006	-1.841	[-0.012, 0.000]	9.30E-02	1.11E-01	AIC=2761.5; R2m=0.014; R2c=0.017
ADA	-0.006	-2.049	[-0.012, -0.000]	4.07E-02	5.02E-02	AIC=1819.9; R2m=0.021; R2c=0.165
TRAIL	-0.006	-3.761	[-0.009, -0.003]	2.10E-04	3.45E-04	AIC=593.5; R2m=0.033; R2c=0.082
MCP-1	-0.006	-3.268	[-0.010, -0.003]	3.75E-03	5.50E-03	AIC=1684.1; R2m=0.018; R2c=0.020
FGF-19	-0.006	-1.497	[-0.015, 0.002]	1.49E-01	1.74E-01	AIC=3348.2; R2m=0.012; R2c=0.017
IL8	-0.007	-1.856	[-0.014, 0.000]	6.41E-02	7.76E-02	AIC=2439.2; R2m=0.011; R2c=0.091
MMP-10	-0.009	-4.150	[-0.013, -0.005]	3.57E-05	6.49E-05	AIC=2087.8; R2m=0.029; R2c=0.029
CCL19	-0.009	-2.687	[-0.016, -0.003]	1.02E-02	1.37E-02	AIC=2565.1; R2m=0.013; R2c=0.029
CD40	-0.010	-3.911	[-0.015, -0.005]	9.77E-05	1.69E-04	AIC=1438.0; R2m=0.040; R2c=0.305
CX3CL1	-0.010	-4.393	[-0.014, -0.005]	2.57E-05	4.80E-05	AIC=1386.6; R2m=0.042; R2c=0.070
DNER	-0.011	-8.620	[-0.013, -0.008]	5.38E-12	2.32E-11	AIC=271.4; R2m=0.120; R2c=0.133
SCF	-0.012	-8.323	[-0.015, -0.009]	2.40E-16	1.84E-15	AIC=1194.4; R2m=0.066; R2c=0.066
CD244	-0.012	-6.076	[-0.016, -0.008]	2.32E-09	6.96E-09	AIC=940.3; R2m=0.079; R2c=0.168
4E-BP1	-0.014	-2.781	[-0.024, -0.004]	5.96E-03	8.22E-03	AIC=3195.9; R2m=0.014; R2c=0.059
IL7	-0.014	-4.615	[-0.020, -0.008]	4.43E-06	9.55E-06	AIC=1936.7; R2m=0.043; R2c=0.262
CD6	-0.014	-4.022	[-0.021, -0.007]	6.14E-05	1.09E-04	AIC=2279.0; R2m=0.019; R2c=0.531
CD5	-0.015	-7.311	[-0.019, -0.011]	5.26E-13	2.79E-12	AIC=960.1; R2m=0.085; R2c=0.308
IFN-γ	-0.015	-3.446	[-0.024, -0.007]	2.88E-03	4.41E-03	AIC=3490.5; R2m=0.020; R2c=0.024

CXCL1	-0.016	-2.924	[-0.026, -0.005]	3.53E-03	5.29E-03	AIC=3193.6; R2m=0.018; R2c=0.318
CASP-8	-0.016	-3.291	[-0.026, -0.006]	1.03E-03	1.62E-03	AIC=2989.9; R2m=0.024; R2c=0.311
ST1A1	-0.019	-2.786	[-0.032, -0.005]	5.42E-03	7.63E-03	AIC=3707.3; R2m=0.011; R2c=0.387
CCL11	-0.019	-8.239	[-0.024, -0.015]	2.50E-11	9.57E-11	AIC=1612.5; R2m=0.106; R2c=0.125
TNFSF14	-0.021	-6.340	[-0.027, -0.014]	3.52E-10	1.16E-09	AIC=2087.9; R2m=0.076; R2c=0.257
CCL23	-0.022	-7.969	[-0.027, -0.016]	1.21E-14	8.37E-14	AIC=1717.3; R2m=0.109; R2c=0.181
CXCL6	-0.022	-4.675	[-0.031, -0.013]	3.37E-06	7.51E-06	AIC=2926.8; R2m=0.048; R2c=0.225
MCP-2	-0.024	-6.251	[-0.031, -0.016]	3.99E-09	1.14E-08	AIC=2616.4; R2m=0.069; R2c=0.102
EN-RAGE	-0.024	-6.463	[-0.031, -0.017]	1.63E-10	5.62E-10	AIC=2400.1; R2m=0.070; R2c=0.251
IL-12B	-0.025	-7.865	[-0.031, -0.019]	4.84E-14	3.03E-13	AIC=2105.5; R2m=0.108; R2c=0.168
STAMBP	-0.025	-4.690	[-0.036, -0.015]	3.10E-06	7.14E-06	AIC=3249.2; R2m=0.038; R2c=0.247
AXIN1	-0.029	-4.330	[-0.042, -0.016]	1.62E-05	3.10E-05	AIC=3695.1; R2m=0.024; R2c=0.483
TWEAK	-0.030	-15.969	[-0.034, -0.027]	3.13E-35	4.32E-34	AIC=981.8; R2m=0.327; R2c=0.354
TRANCE	-0.032	-12.443	[-0.037, -0.027]	1.87E-13	1.07E-12	AIC=1925.1; R2m=0.202; R2c=0.212
SIRT2	-0.033	-4.750	[-0.046, -0.019]	2.31E-06	5.49E-06	AIC=3810.3; R2m=0.036; R2c=0.300
MCP-4	-0.037	-8.908	[-0.045, -0.029]	3.64E-18	3.14E-17	AIC=2657.3; R2m=0.137; R2c=0.254
CD8A	-0.051	-12.409	[-0.059, -0.043]	7.29E-32	8.38E-31	AIC=2650.3; R2m=0.225; R2c=0.308
CXCL5	-0.060	-7.137	[-0.076, -0.043]	1.73E-12	7.97E-12	AIC=4251.7; R2m=0.081; R2c=0.346

Supplementary Table 8. Spline models for continuous gestational age. Abbreviations: AIC, Akaike information criterion; FDR, false discovery rate; NPX, Normalized Protein eXpression.

Protein	F	p (uncorrected)	p (FDR-adjusted)	Predicted ΔNPX (late–early)	Model fit
CCL28	566.594	1.76E-122	4.04E-121	3.145	AIC=3027.6; R2m=0.334; R2c=0.334
LIF-R	697.886	6.03E-151	2.08E-149	2.371	AIC=1725.4; R2m=0.512; R2c=0.526
PD-L1	932.475	8.00E-202	5.52E-200	2.177	AIC=1611.4; R2m=0.598; R2c=0.618
FGF-21	45.152	8.59E-10	1.92E-09	1.238	AIC=4751.4; R2m=0.084; R2c=0.109
uPA	535.116	1.17E-115	2.02E-114	1.208	AIC=830.1; R2m=0.548; R2c=0.593
LAP-TGF-β-1	289.270	2.09E-62	2.40E-61	1.083	AIC=1617.2; R2m=0.400; R2c=0.473
OPG	237.216	3.81E-51	3.75E-50	1.000	AIC=1977.4; R2m=0.236; R2c=0.239
CDCP1	189.409	8.19E-41	7.06E-40	0.894	AIC=1980.6; R2m=0.158; R2c=0.159
FGF-23	130.941	3.39E-28	1.80E-27	0.789	AIC=4277.1; R2m=0.175; R2c=0.179
HGF	102.024	5.70E-22	2.62E-21	0.707	AIC=1260.4; R2m=0.150; R2c=0.189
IL-17C	32.868	3.43E-07	5.92E-07	0.614	AIC=2366.0; R2m=0.070; R2c=0.138
MMP-1	6.326	9.68E-02	1.06E-01	0.501	AIC=3493.4; R2m=0.012; R2c=0.184
IL-10RB	157.903	5.19E-34	3.58E-33	0.474	AIC=230.4; R2m=0.242; R2c=0.287
OSM	5.595	1.33E-01	1.43E-01	0.429	AIC=3111.7; R2m=0.017; R2c=0.096
IL18	28.402	2.99E-06	4.80E-06	0.381	AIC=2055.0; R2m=0.046; R2c=0.073
TGF-α	35.268	1.07E-07	1.94E-07	0.352	AIC=1124.1; R2m=0.028; R2c=0.248
CCL25	36.116	7.08E-08	1.32E-07	0.315	AIC=1939.3; R2m=0.036; R2c=0.036
VEGFA	55.849	4.52E-12	1.25E-11	0.311	AIC=243.6; R2m=0.102; R2c=0.202
IL-15RA	38.711	2.00E-08	3.94E-08	0.307	AIC=491.3; R2m=0.078; R2c=0.116
CSF-1	50.551	6.10E-11	1.62E-10	0.237	AIC=-392.3; R2m=0.075; R2c=0.139
Flt3L	7.236	6.47E-02	7.32E-02	0.221	AIC=1193.2; R2m=0.036; R2c=0.047
IL-18R1	21.127	9.91E-05	1.45E-04	0.160	AIC=1480.5; R2m=0.047; R2c=0.071
IL6	18.613	3.29E-04	4.54E-04	0.134	AIC=2667.6; R2m=0.031; R2c=0.096
IL8	42.559	3.05E-09	6.49E-09	0.116	AIC=2404.9; R2m=0.039; R2c=0.119
IL10	3.212	3.60E-01	3.65E-01	0.110	AIC=2345.4; R2m=0.011; R2c=0.011
TNF	2.577	4.62E-01	4.62E-01	0.109	AIC=1773.9; R2m=0.006; R2c=0.033
CST5	14.450	2.35E-03	3.01E-03	0.030	AIC=1809.8; R2m=0.025; R2c=0.050
CCL3	9.086	2.82E-02	3.35E-02	0.020	AIC=2471.1; R2m=0.022; R2c=0.063
CXCL11	6.977	7.26E-02	8.08E-02	0.015	AIC=3208.3; R2m=0.007; R2c=0.152
CCL4	4.438	2.18E-01	2.24E-01	-0.020	AIC=2005.8; R2m=0.008; R2c=0.069
FGF-19	5.195	1.58E-01	1.68E-01	-0.035	AIC=3350.5; R2m=0.014; R2c=0.017
MCP-1	36.435	6.06E-08	1.16E-07	-0.112	AIC=1661.4; R2m=0.041; R2c=0.050
NT-3	47.891	2.25E-10	5.34E-10	-0.128	AIC=1678.8; R2m=0.049; R2c=0.050
MMP-10	18.810	2.99E-04	4.21E-04	-0.156	AIC=2090.2; R2m=0.030; R2c=0.030
CXCL9	14.542	2.25E-03	2.93E-03	-0.184	AIC=2754.5; R2m=0.023; R2c=0.027
ADA	12.384	6.18E-03	7.61E-03	-0.205	AIC=1815.8; R2m=0.025; R2c=0.174

TNFB	11.515	9.24E-03	1.12E-02	-0.234	AIC=1302.7; R2m=0.013; R2c=0.027
TRAIL	45.139	8.65E-10	1.92E-09	-0.282	AIC=567.4; R2m=0.046; R2c=0.097
TNFRSF9	70.946	2.68E-15	8.80E-15	-0.294	AIC=1067.9; R2m=0.078; R2c=0.120
CCL20	4.749	1.91E-01	2.00E-01	-0.297	AIC=3247.3; R2m=0.019; R2c=0.020
IL7	33.783	2.20E-07	3.89E-07	-0.297	AIC=1928.6; R2m=0.048; R2c=0.277
CX3CL1	32.708	3.71E-07	6.25E-07	-0.314	AIC=1376.6; R2m=0.047; R2c=0.091
DNER	79.819	3.36E-17	1.36E-16	-0.346	AIC=262.4; R2m=0.117; R2c=0.138
CD40	39.714	1.23E-08	2.49E-08	-0.358	AIC=1417.9; R2m=0.049; R2c=0.287
SCF	73.982	5.99E-16	2.17E-15	-0.380	AIC=1178.2; R2m=0.079; R2c=0.083
CXCL6	49.398	1.07E-10	2.74E-10	-0.387	AIC=2904.2; R2m=0.060; R2c=0.245
4E-BP1	15.333	1.55E-03	2.10E-03	-0.398	AIC=3192.1; R2m=0.017; R2c=0.055
CCL19	26.438	7.72E-06	1.21E-05	-0.415	AIC=2550.8; R2m=0.027; R2c=0.058
CCL11	171.933	4.87E-37	3.73E-36	-0.445	AIC=1522.7; R2m=0.158; R2c=0.174
CD6	31.608	6.33E-07	1.04E-06	-0.454	AIC=2267.9; R2m=0.019; R2c=0.532
CXCL1	9.037	2.88E-02	3.37E-02	-0.477	AIC=3197.1; R2m=0.020; R2c=0.323
CASP-8	13.898	3.05E-03	3.82E-03	-0.507	AIC=2990.9; R2m=0.023; R2c=0.316
CD244	42.524	3.11E-09	6.49E-09	-0.535	AIC=938.1; R2m=0.069; R2c=0.142
TNFSF14	72.423	1.29E-15	4.46E-15	-0.538	AIC=2061.3; R2m=0.083; R2c=0.273
CD5	66.208	2.77E-14	8.68E-14	-0.596	AIC=952.0; R2m=0.069; R2c=0.299
IFN-γ	15.006	1.81E-03	2.40E-03	-0.680	AIC=3491.9; R2m=0.019; R2c=0.019
EN-RAGE	48.485	1.68E-10	4.14E-10	-0.750	AIC=2397.6; R2m=0.066; R2c=0.249
MCP-2	61.189	3.27E-13	9.82E-13	-0.807	AIC=2598.9; R2m=0.072; R2c=0.118
ST1A1	8.634	3.46E-02	3.98E-02	-0.826	AIC=3710.4; R2m=0.010; R2c=0.384
IL-12B	96.208	1.02E-20	4.38E-20	-0.884	AIC=2076.5; R2m=0.104; R2c=0.147
CCL23	74.547	4.53E-16	1.74E-15	-0.942	AIC=1710.6; R2m=0.092; R2c=0.171
STAMBP	26.000	9.54E-06	1.46E-05	-0.979	AIC=3249.2; R2m=0.032; R2c=0.234
TWEAK	339.908	2.29E-73	3.15E-72	-1.006	AIC=910.7; R2m=0.326; R2c=0.367
MCP-4	124.382	8.78E-27	4.33E-26	-1.087	AIC=2620.1; R2m=0.139; R2c=0.275
AXIN1	19.541	2.11E-04	3.04E-04	-1.127	AIC=3698.3; R2m=0.021; R2c=0.476
TRANCE	152.507	7.59E-33	4.36E-32	-1.174	AIC=1917.2; R2m=0.195; R2c=0.215
SIRT2	24.809	1.69E-05	2.54E-05	-1.250	AIC=3812.1; R2m=0.031; R2c=0.291
CXCL5	57.857	1.69E-12	4.85E-12	-1.510	AIC=4249.3; R2m=0.094; R2c=0.367
CD8A	157.400	6.67E-34	4.19E-33	-1.776	AIC=2651.4; R2m=0.215; R2c=0.295

Supplementary Table 9. Generalized additive models for continuous gestational age. Abbreviations: AIC, Akaike information criterion; EDF, estimated degrees of freedom; FDR, false discovery rate; NPX, Normalized Protein eXpression.

Protein	EDF	F	p (uncorrected)	p (FDR-adjusted)	Predicted ΔNPX (late–early)	Model fit
CCL28	3.610	124.511	7.68E-97	1.77E-95	2.824	AIC=3028.3; R2m=0.330; R2c=0.330
LIF-R	5.970	102.062	4.83E-117	1.67E-115	2.822	AIC=1686.3; R2m=0.559; R2c=0.559
PD-L1	5.370	138.443	1.25E-139	8.64E-138	2.407	AIC=1598.5; R2m=0.643; R2c=0.643
FGF-21	3.600	9.849	1.77E-08	3.31E-08	1.522	AIC=4741.4; R2m=0.085; R2c=0.085
LAP-TGF-β-1	4.810	47.897	1.12E-51	1.29E-50	1.305	AIC=1600.5; R2m=0.500; R2c=0.500
uPA	4.290	97.216	2.60E-89	4.48E-88	1.251	AIC=816.3; R2m=0.629; R2c=0.629
OPG	2.300	65.879	1.73E-37	1.71E-36	1.166	AIC=1977.3; R2m=0.233; R2c=0.233
FGF-23	4.650	20.758	4.39E-22	2.16E-21	1.021	AIC=4278.0; R2m=0.172; R2c=0.172
CDCP1	1.000	171.510	1.01E-36	8.69E-36	1.018	AIC=1976.8; R2m=0.155; R2c=0.155
IL-17C	1.380	18.713	1.35E-06	2.21E-06	0.696	AIC=2350.5; R2m=0.136; R2c=0.136
HGF	4.430	17.611	4.73E-18	2.04E-17	0.692	AIC=1250.3; R2m=0.154; R2c=0.154
IL-10RB	3.300	36.415	2.70E-29	1.69E-28	0.530	AIC=220.5; R2m=0.260; R2c=0.260
IL18	2.260	8.768	3.57E-05	5.36E-05	0.441	AIC=2046.2; R2m=0.072; R2c=0.072
AXIN1	6.780	6.887	9.10E-09	1.79E-08	0.370	AIC=3640.2; R2m=0.325; R2c=0.325
VEGFA	1.000	51.555	1.24E-12	3.57E-12	0.369	AIC=224.7; R2m=0.202; R2c=0.202
CSF-1	4.490	10.771	6.21E-11	1.53E-10	0.316	AIC=-416.5; R2m=0.139; R2c=0.139
IL-15RA	2.020	14.421	4.47E-08	7.90E-08	0.311	AIC=479.7; R2m=0.100; R2c=0.100
TGF-α	4.620	5.636	1.48E-05	2.27E-05	0.286	AIC=1104.7; R2m=0.199; R2c=0.199
IL8	4.460	9.453	1.77E-09	4.07E-09	0.285	AIC=2382.0; R2m=0.082; R2c=0.082
MMP-1	1.910	1.452	2.54E-01	2.74E-01	0.269	AIC=3474.6; R2m=0.126; R2c=0.126
CCL3	1.000	4.094	4.31E-02	4.88E-02	0.263	AIC=2461.0; R2m=0.038; R2c=0.038
IL-18R1	2.480	5.393	1.01E-03	1.37E-03	0.252	AIC=1473.4; R2m=0.055; R2c=0.055
CCL25	3.320	8.374	1.02E-06	1.75E-06	0.183	AIC=1938.1; R2m=0.034; R2c=0.034
Flt3L	1.000	5.619	1.79E-02	2.06E-02	0.161	AIC=1184.1; R2m=0.050; R2c=0.050

IL7	5.710	7.639	6.85E-09	1.39E-08	0.151	AIC=1890.2; R2m=0.169; R2c=0.169
IL6	4.280	4.136	7.30E-04	1.01E-03	0.141	AIC=2650.9; R2m=0.074; R2c=0.074
STAMBP	6.890	8.042	1.47E-10	3.50E-10	0.135	AIC=3194.3; R2m=0.130; R2c=0.130
CXCL6	5.190	10.003	3.31E-11	8.47E-11	0.110	AIC=2872.5; R2m=0.122; R2c=0.122
ST1A1	5.960	3.864	3.51E-04	4.94E-04	0.106	AIC=3668.8; R2m=0.258; R2c=0.258
OSM	1.000	0.270	6.04E-01	6.13E-01	0.092	AIC=3098.3; R2m=0.073; R2c=0.073
CST5	3.030	3.877	5.38E-03	6.63E-03	0.075	AIC=1800.2; R2m=0.024; R2c=0.024
SIRT2	6.680	7.224	3.53E-09	7.86E-09	0.074	AIC=3761.8; R2m=0.161; R2c=0.161
IL10	1.600	0.522	5.49E-01	5.65E-01	0.065	AIC=2342.9; R2m=0.010; R2c=0.010
TNF	1.440	0.236	6.87E-01	6.87E-01	0.036	AIC=1764.6; R2m=0.025; R2c=0.025
EN-RAGE	6.400	10.222	3.27E-13	1.08E-12	-0.053	AIC=2352.1; R2m=0.230; R2c=0.230
CCL4	1.830	1.321	2.95E-01	3.13E-01	-0.085	AIC=1992.5; R2m=0.042; R2c=0.042
CD40	5.200	8.108	6.25E-09	1.31E-08	-0.086	AIC=1386.2; R2m=0.181; R2c=0.181
CXCL11	2.190	2.064	1.40E-01	1.56E-01	-0.087	AIC=3190.2; R2m=0.088; R2c=0.088
NT-3	3.670	10.250	6.11E-09	1.31E-08	-0.093	AIC=1677.4; R2m=0.047; R2c=0.047
MCP-1	3.510	8.475	1.17E-06	1.98E-06	-0.099	AIC=1654.8; R2m=0.043; R2c=0.043
CCL20	2.080	0.948	3.07E-01	3.21E-01	-0.131	AIC=3245.8; R2m=0.017; R2c=0.017
ADA	2.830	3.350	1.34E-02	1.60E-02	-0.158	AIC=1795.8; R2m=0.120; R2c=0.120
TNFB	2.160	3.651	1.68E-02	1.97E-02	-0.175	AIC=1296.8; R2m=0.021; R2c=0.021
FGF-19	1.000	1.465	2.25E-01	2.47E-01	-0.200	AIC=3345.2; R2m=0.016; R2c=0.016
TNFRSF9	3.740	14.717	3.49E-13	1.09E-12	-0.226	AIC=1058.3; R2m=0.085; R2c=0.085
TRAIL	3.190	10.555	2.34E-08	4.24E-08	-0.252	AIC=556.6; R2m=0.073; R2c=0.073
CXCL9	2.550	4.105	5.84E-03	7.06E-03	-0.273	AIC=2751.6; R2m=0.023; R2c=0.023
TNFSF14	5.520	12.436	8.33E-15	3.19E-14	-0.280	AIC=2032.7; R2m=0.180; R2c=0.180
MMP-10	1.000	17.127	3.79E-05	5.57E-05	-0.306	AIC=2085.8; R2m=0.028; R2c=0.028
CX3CL1	2.660	9.213	2.97E-06	4.76E-06	-0.329	AIC=1365.3; R2m=0.069; R2c=0.069
DNER	3.010	19.814	6.11E-15	2.48E-14	-0.370	AIC=253.6; R2m=0.159; R2c=0.159
4E-BP1	2.810	4.317	3.39E-03	4.42E-03	-0.392	AIC=3180.4; R2m=0.034; R2c=0.034

CCL19	2.900	6.982	5.20E-05	7.47E-05	-0.410	AIC=2541.4; R2m=0.034; R2c=0.034
SCF	3.210	16.929	1.95E-13	6.74E-13	-0.462	AIC=1175.8; R2m=0.082; R2c=0.082
CD244	3.500	10.306	1.00E-08	1.92E-08	-0.490	AIC=922.1; R2m=0.070; R2c=0.070
CD6	3.370	7.538	4.55E-06	7.13E-06	-0.515	AIC=2236.9; R2m=0.455; R2c=0.455
CASP-8	2.040	4.951	3.62E-03	4.62E-03	-0.532	AIC=2965.7; R2m=0.260; R2c=0.260
CD5	2.750	18.126	8.84E-13	2.65E-12	-0.550	AIC=931.0; R2m=0.246; R2c=0.246
CXCL1	1.000	8.638	3.32E-03	4.40E-03	-0.552	AIC=3169.7; R2m=0.193; R2c=0.193
CCL11	4.660	29.359	2.17E-31	1.50E-30	-0.583	AIC=1517.7; R2m=0.167; R2c=0.167
IFN-γ	1.390	8.626	3.86E-03	4.85E-03	-0.588	AIC=3488.4; R2m=0.019; R2c=0.019
MCP-2	3.120	15.243	7.32E-12	2.02E-11	-0.774	AIC=2586.4; R2m=0.102; R2c=0.102
IL-12B	3.480	22.445	4.67E-19	2.15E-18	-0.784	AIC=2063.4; R2m=0.100; R2c=0.100
TWEAK	6.010	49.524	1.01E-62	1.39E-61	-0.813	AIC=884.2; R2m=0.385; R2c=0.385
CCL23	3.150	17.817	5.44E-14	1.98E-13	-0.868	AIC=1697.3; R2m=0.167; R2c=0.167
MCP-4	3.860	26.349	2.60E-24	1.38E-23	-1.001	AIC=2599.0; R2m=0.230; R2c=0.230
TRANCE	2.570	45.168	4.66E-29	2.68E-28	-1.160	AIC=1909.1; R2m=0.210; R2c=0.210
CXCL5	3.940	12.645	9.65E-12	2.56E-11	-1.331	AIC=4223.2; R2m=0.180; R2c=0.180
CD8A	1.720	71.757	8.75E-32	6.71E-31	-1.781	AIC=2634.2; R2m=0.223; R2c=0.223

Supplementary Table 10. Fetal sex interaction models. Supplementary Tables 10 and 11 show model statistics from generalized additive models (GAMs) testing for gestational age (GA) × categorical modifier (fetal sex or gravidity) interactions. Each protein was modeled with a smooth term for GA and modifier-specific smooths to test for differential associations. The F-statistic and associated numerator (Num. df) and denominator (Den. df) degrees of freedom reflect the joint test comparing the full (interaction) model to a reduced model with a single GA smooth shared across groups. Akaike information criterion values indicate overall model fit. Reported p-values correspond to the uncorrected and false discovery rate-adjusted significance levels for the joint interaction test. NA values indicate proteins for which the full and baseline models yielded indistinguishable fits, resulting in no valid comparison (i.e., no additional variance explained by the modifier-specific smooths). Abbreviations: AIC, Akaike information criterion; FDR, false discovery rate.

Protein	F	Num. df	Den. df	AIC	p (uncorrected)	p (FDR-adjusted)
CD244	5.130	4.050	1138.690	910.920	<0.001	0.026
LAP-TGF-β-1	3.410	4.100	1135.480	1591.330	0.008	0.266
CD8A	7.760	0.400	1141.890	2631.980	0.024	0.500
IL-17C	2.860	3.270	1139.570	2345.940	0.032	0.500
IL18	2.570	3.870	1136.900	2043.140	0.038	0.500
CCL19	3.650	1.060	1139.300	2539.690	0.054	0.540
PD-L1	3.370	1.010	1138.630	1598.520	0.066	0.540
TRANCE	3.740	0.840	1139.600	1908.060	0.060	0.540
NT-3	2.750	1.010	1141.270	1675.690	0.097	0.702
MCP-1	1.880	3.740	1136.520	1654.100	0.117	0.758
VEGFA	0.980	1.000	1141.850	225.790	0.322	0.873
IL6	1.350	2.470	1138.000	2655.210	0.260	0.873
CXCL11	0.680	1.040	1140.040	3191.380	0.414	0.873
TRAIL	2.090	0.970	1139.280	556.460	0.149	0.873
CXCL9	0.720	1.090	1140.600	2753.140	0.409	0.873
OSM	0.560	0.990	1141.860	3099.710	0.454	0.873
CXCL1	0.380	0.990	1141.940	3171.320	0.536	0.873
CCL4	0.350	1.100	1140.830	1994.620	0.574	0.873
MCP-4	0.490	0.980	1138.910	2600.030	0.479	0.873
TNFSF14	0.840	0.960	1138.810	2040.010	0.354	0.873
MMP-1	0.400	0.730	1141.240	3476.000	0.463	0.873
FGF-21	0.270	1.010	1139.180	4744.530	0.603	0.873
IL-10RB	0.250	1.000	1141.830	223.890	0.618	0.873
IL-18R1	0.380	1.030	1139.680	1475.950	0.546	0.873
CXCL5	0.250	1.000	1141.920	4230.100	0.614	0.873
HGF	1.000	1.000	1138.890	1252.220	0.318	0.873
IL-12B	0.310	1.020	1138.700	2064.750	0.580	0.873
MMP-10	1.140	1.000	1147.000	2086.650	0.286	0.873
TNF	1.840	0.990	1141.890	1763.770	0.175	0.873
CCL23	0.500	0.890	1139.240	1699.030	0.461	0.873
CCL3	0.720	1.000	1141.600	2462.730	0.396	0.873
Flt3L	0.930	1.000	1142.250	1185.470	0.334	0.873
CXCL6	1.470	3.530	1136.070	2876.250	0.215	0.873
4E-BP1	0.810	0.960	1139.530	3182.130	0.363	0.873
CCL28	0.340	0.990	1144.130	3030.090	0.555	0.873
CD40	0.640	1.000	1138.640	1389.140	0.424	0.873
IFN-γ	0.340	1.000	1143.210	3489.890	0.559	0.873

FGF-19	0.700	1.010	1142.790	3346.970	0.403	0.873
MCP-2	0.910	1.000	1139.360	2587.440	0.340	0.873
CCL25	1.380	1.050	1144.170	1938.500	0.241	0.873
CX3CL1	0.370	1.010	1139.470	1366.990	0.547	0.873
TNFRSF9	0.510	0.990	1138.990	1059.480	0.474	0.873
TWEAK	0.740	1.010	1138.920	897.810	0.391	0.873
CCL20	1.730	1.580	1146.410	3245.300	0.185	0.873
STAMBP	0.280	1.000	1138.640	3215.070	0.599	0.873
TNFB	0.610	1.080	1140.400	1298.980	0.446	0.873
IL8	0.110	0.960	1138.620	2382.260	0.724	0.898
uPA	0.090	1.000	1138.840	816.810	0.764	0.898
CST5	0.090	1.020	1139.110	1802.580	0.767	0.898
SCF	0.080	0.980	1140.290	1177.400	0.773	0.898
CCL11	0.120	1.000	1138.440	1519.290	0.728	0.898
LIF-R	0.110	1.000	1138.700	1690.030	0.739	0.898
DNER	0.100	1.010	1139.630	256.110	0.753	0.898
CASP-8	0.110	1.030	1141.030	2967.900	0.753	0.898
ADA	0.200	1.010	1139.630	1797.860	0.655	0.898
CSF-1	0.090	1.000	1138.390	-414.890	0.764	0.898
EN-RAGE	0.060	1.000	1138.620	2367.060	0.807	0.921
CDCP1	0.010	0.950	1146.770	1977.680	0.894	0.965
IL7	0.020	1.000	1138.640	1898.270	0.893	0.965
OPG	0.010	1.000	1142.290	1980.220	0.908	0.965
CD6	0.020	1.010	1139.540	2239.660	0.876	0.965
TGF-α	0.010	1.000	1138.740	1107.600	0.930	0.965
IL-15RA	0.000	0.860	1140.560	481.740	0.965	0.965
IL10	0.000	1.000	1147.000	2343.340	0.949	0.965
ST1A1	0.000	1.000	1141.940	3683.410	0.960	0.965
AXIN1	NA	NA	NA	NA	NA	NA
FGF-23	NA	NA	NA	NA	NA	NA
CD5	NA	NA	NA	NA	NA	NA
SIRT2	NA	NA	NA	NA	NA	NA

Supplemental Table 11. Sex differences in CD244 expression by trimester. To further characterize male-female differences within each trimester for proteins demonstrating significant gestational age x fetal sex interactions, we conducted trimester-stratified linear mixed-effects models with random intercepts for cohort, adjusting for gravidity and maternal age. In the first trimester, there was a trend-level sex difference in CD244 expression ($B = -0.11$, $p = 0.097$; $N = 103$). In the second trimester, a significant male-female difference was observed ($B = -0.21$, $p < 0.001$; $N = 169$). In the third trimester, no significant sex difference was detected ($B = -0.03$, $p = 0.24$; $N = 882$). These trimester-specific results are consistent with our findings from our continuous model indicating sex-related differences that vary across gestation, peaking in the 2nd trimester.

	**N**	**B estimate**	**t statistic**	**p-value**
**1st trimester**	103	-0.11	-1.68	0.097
**2nd trimester**	169	-0.21	-3.76	<0.001
**3rd trimester**	882	-0.03	-1.17	0.24

Supplementary Table 12. Gravidity interaction models. Each protein was modeled with a smooth term for GA and modifier-specific smooths to test for differential associations. The F-statistic and associated numerator (Num. df) and denominator (Den. df) degrees of freedom reflect the joint test comparing the full (interaction) model to a reduced model with a single GA smooth shared across groups. Akaike information criterion values indicate overall model fit. Reported p-values correspond to the uncorrected and false discovery rate-adjusted significance levels for the joint interaction test. NA values indicate proteins for which the full and baseline models yielded indistinguishable fits, resulting in no valid comparison (i.e., no additional variance explained by the modifier-specific smooths). Abbreviations: AIC, Akaike information criterion; FDR, false discovery rate.

Protein	F	Num. df	Den. df	AIC	p (uncorrected)	p (FDR-adjusted)
CST5	26.500	0.300	1139.820	1792.200	0.001	0.050
SIRT2	4.030	4.810	1137.890	3776.720	0.001	0.050
ADA	3.070	4.650	1136.000	1789.980	0.011	0.255
CDCP1	3.470	1.720	1145.990	1973.380	0.038	0.432
IL-15RA	2.980	2.580	1138.840	477.030	0.038	0.432
CASP-8	2.710	3.930	1138.140	2962.560	0.030	0.432
LAP-TGF-β-1	2.360	1.990	1137.590	1597.440	0.095	0.467
IL6	2.960	1.880	1138.600	2652.300	0.056	0.467
CD6	2.090	3.410	1137.140	2236.570	0.092	0.467
MMP-1	2.160	2.760	1139.200	3473.870	0.096	0.467
CCL19	2.650	1.820	1138.540	2540.190	0.076	0.467
CCL23	2.990	1.420	1138.710	1696.390	0.068	0.467
DNER	2.900	1.970	1138.660	252.440	0.056	0.467
CCL20	2.170	3.550	1144.440	3243.830	0.078	0.467
MMP-10	2.130	2.000	1146.000	2085.510	0.119	0.539
CXCL11	1.810	2.750	1138.330	3190.110	0.149	0.545
Flt3L	1.940	1.660	1141.580	1184.450	0.152	0.545
4E-BP1	2.060	1.820	1138.680	3180.780	0.133	0.545
FGF-19	1.750	3.620	1140.170	3345.740	0.143	0.545
CD244	1.530	5.080	1137.670	925.830	0.178	0.547
TNFSF14	1.640	3.400	1136.360	2039.250	0.171	0.547
NT-3	1.640	2.670	1139.610	1677.040	0.183	0.547
STAMBP	1.700	1.900	1137.740	3213.430	0.185	0.547
IL-18R1	1.620	1.950	1138.760	1474.860	0.199	0.565
IL8	1.540	2.140	1137.430	2380.910	0.214	0.576
TNFB	1.520	1.940	1139.540	1298.300	0.220	0.576
LIF-R	1.280	2.040	1137.670	1689.500	0.278	0.675
IL-10RB	1.270	5.020	1137.810	224.560	0.274	0.675
CD8A	1.200	1.400	1140.890	2635.420	0.290	0.680
SCF	1.140	2.690	1138.570	1177.230	0.328	0.697
IL18	1.120	1.960	1138.810	2048.040	0.327	0.697
CD5	1.190	2.810	1137.690	932.360	0.311	0.697
TGF-α	0.970	2.000	1137.740	1107.630	0.381	0.728
TNF	0.940	1.860	1141.020	1765.430	0.386	0.728
IFN-γ	1.040	2.200	1142.020	3490.270	0.358	0.728
CX3CL1	0.870	1.400	1139.070	1366.860	0.385	0.728
IL7	0.860	2.010	1137.630	1898.510	0.425	0.729
CXCL9	0.840	2.300	1139.390	2754.370	0.445	0.729
CCL11	0.920	2.800	1136.640	1519.850	0.425	0.729
CCL28	0.800	1.990	1143.130	3030.820	0.447	0.729

TWEAK	1.010	3.750	1136.180	899.410	0.398	0.729
ST1A1	0.800	2.000	1140.940	3683.790	0.450	0.729
VEGFA	0.780	2.000	1140.840	227.150	0.461	0.729
CCL3	0.730	2.080	1140.510	2464.000	0.490	0.757
CXCL1	0.660	2.000	1140.930	3172.350	0.515	0.762
TNFRSF9	0.670	1.990	1137.990	1060.510	0.511	0.762
OPG	0.680	2.560	1140.730	1981.410	0.544	0.776
FGF-23	0.390	1.070	1142.760	4277.990	0.548	0.776
IL-17C	0.560	1.990	1140.850	2352.590	0.568	0.783
PD-L1	0.550	1.960	1137.670	1602.720	0.576	0.783
uPA	0.450	1.960	1137.880	817.880	0.634	0.798
MCP-1	0.410	1.570	1138.690	1657.000	0.613	0.798
HGF	0.460	1.890	1137.990	1254.080	0.624	0.798
CCL25	0.460	1.870	1143.350	1940.740	0.621	0.798
CCL4	0.330	2.220	1139.710	1996.380	0.738	0.912
TRAIL	0.220	1.900	1138.350	559.920	0.791	0.945
FGF-21	0.180	1.670	1138.520	4745.510	0.792	0.945
TRANCE	0.180	1.760	1138.680	1912.580	0.809	0.948
CXCL6	0.170	2.030	1137.570	2879.120	0.843	0.955
CD40	0.190	2.020	1137.620	1391.360	0.831	0.955
OSM	0.090	1.960	1140.880	3102.010	0.914	0.972
EN-RAGE	0.090	2.010	1137.610	2368.920	0.915	0.972
MCP-2	0.080	1.860	1138.500	2589.860	0.908	0.972
CSF-1	0.100	2.030	1137.360	-413.080	0.909	0.972
IL10	0.060	2.000	1146.000	2345.220	0.944	0.988
MCP-4	0.020	2.020	1137.870	2602.510	0.977	0.991
IL-12B	0.020	1.840	1137.880	2066.600	0.969	0.991
CXCL5	0.010	2.000	1140.920	4232.330	0.992	0.992
AXIN1	NA	NA	NA	NA	NA	NA

Supplementary Table 13. Maternal age interaction models. This table shows model statistics from generalized additive models testing for continuous modification of gestational age (GA) trajectories by maternal age at blood draw. Each protein was modeled with main-effect smooths for GA and maternal age and a tensor-product interaction smooth to capture continuous modification of GA–protein relationships by maternal age. The estimated degrees of freedom and F-statistic describe the shape and strength of the interaction smooth, respectively, and Akaike information criterion values indicate overall model fit. Reported p-values correspond to the uncorrected and false discovery rate-adjusted significance levels for the maternal age interaction term. Abbreviations: AIC, Akaike information criterion; EDF, estimated degrees of freedom; FDR, false discovery rate.

Protein	EDF	F	AIC	p (uncorrected)	p (FDR-adjusted)
TGF-α	1.000	8.150	1098.960	0.004	0.262
IL10	7.690	2.190	2331.560	0.011	0.262
TNF	1.000	6.420	1760.690	0.011	0.262
VEGFA	2.180	2.860	221.070	0.036	0.452
uPA	1.000	3.990	813.060	0.046	0.452
TNFSF14	3.940	2.270	2032.760	0.035	0.452
HGF	1.000	4.200	1248.370	0.041	0.452
TRAIL	1.000	3.480	555.870	0.062	0.473
OSM	1.600	2.550	3096.500	0.094	0.473
CD6	1.480	2.140	2235.670	0.080	0.473
IL-10RB	1.630	2.590	218.750	0.092	0.473
Flt3L	2.370	2.080	1182.360	0.093	0.473
DNER	4.650	1.840	252.220	0.089	0.473
CSF-1	3.450	1.830	-419.140	0.096	0.473
EN-RAGE	1.830	2.050	2363.110	0.103	0.474
IL8	1.000	1.820	2381.120	0.178	0.543
CDCP1	1.000	1.470	1977.180	0.225	0.543
AXIN1	3.440	1.730	3652.490	0.127	0.543
CST5	1.000	1.430	1801.940	0.231	0.543
CXCL1	5.270	1.320	3168.590	0.233	0.543
FGF-23	1.000	2.030	4276.180	0.154	0.543
FGF-21	1.470	2.470	4743.640	0.147	0.543
IL-18R1	4.340	1.700	1472.010	0.195	0.543
CXCL6	1.650	1.470	2875.280	0.217	0.543
SIRT2	4.790	1.530	3775.140	0.152	0.543
IFN-γ	1.000	1.840	3488.130	0.176	0.543
MCP-2	1.000	1.410	2586.770	0.236	0.543
STAMBP	5.010	1.480	3211.740	0.169	0.543
ADA	2.860	1.480	1794.440	0.202	0.543
TNFB	1.510	2.050	1296.640	0.205	0.543
CD5	1.210	0.960	931.080	0.267	0.576
TNFRSF9	1.000	1.230	1057.610	0.267	0.576
IL7	5.240	1.080	1898.090	0.378	0.602
IL6	1.330	1.430	2656.200	0.333	0.602
CXCL11	1.000	0.910	3191.120	0.340	0.602
CXCL9	1.320	0.650	2751.500	0.366	0.602
MCP-4	1.010	0.760	2599.800	0.384	0.602
TRANCE	1.810	0.970	1911.170	0.357	0.602
CCL3	1.000	0.800	2463.660	0.369	0.602
4E-BP1	3.820	1.090	3182.040	0.361	0.602

FGF-19	1.000	0.850	3346.590	0.357	0.602
TWEAK	4.560	1.060	899.020	0.381	0.602
CCL20	4.570	1.100	3245.420	0.342	0.602
ST1A1	3.710	1.060	3681.940	0.382	0.602
LIF-R	1.000	0.680	1689.120	0.410	0.629
LAP-TGF-β-1	2.550	0.750	1599.380	0.444	0.645
CCL4	1.000	0.610	1994.000	0.435	0.645
IL18	1.000	0.570	2047.270	0.451	0.645
IL-12B	1.000	0.480	2064.050	0.486	0.645
MMP-10	2.630	0.650	2086.790	0.470	0.645
CASP-8	4.320	0.900	2968.700	0.485	0.645
CX3CL1	1.000	0.520	1368.100	0.472	0.645
CD40	1.600	0.790	1389.770	0.499	0.650
MMP-1	1.740	0.530	3476.390	0.541	0.692
CD244	1.000	0.240	923.750	0.627	0.713
IL-17C	2.220	0.620	2353.040	0.613	0.713
SCF	1.340	0.620	1177.190	0.611	0.713
CCL11	1.990	0.840	1518.900	0.620	0.713
IL-15RA	1.000	0.290	481.240	0.591	0.713
PD-L1	1.000	0.290	1601.540	0.591	0.713
CCL23	2.670	0.680	1700.090	0.630	0.713
MCP-1	1.970	0.360	1658.080	0.689	0.739
CCL19	2.090	0.430	2545.610	0.677	0.739
CCL25	1.460	0.400	1940.100	0.697	0.739
NT-3	1.000	0.180	1675.710	0.676	0.739
CXCL5	3.420	0.640	4226.420	0.721	0.754
CD8A	1.400	0.370	2636.730	0.736	0.758
CCL28	1.580	0.170	3030.720	0.838	0.850
OPG	1.000	0.010	1977.530	0.920	0.920

2 Supplementary References

1. National Center for Biotechnology Information. Gene. https://www.ncbi.nlm.nih.gov/gene/ [Accessed December 3, 2025].

2. Zhang S, Ding J, Zhang Y, Liu S, Yang J, Yin T. Regulation and function of chemokines at the maternal–fetal interface. Front Cell Dev Biol (2022) 10:826053.

3. Lin Z, Shi JL, Chen M, Zheng ZM, Li MQ, Shao J. CCL2: an important cytokine in normal and pathological pregnancies: a review. Front Immunol (2023) 13:1053457.

4. Liu H, Liu A, Kaminga AC, McDonald J, Wen SW, Pan X. Chemokines in gestational diabetes mellitus. Front Immunol (2022) 13:705852.

5. Camacho-Arroyo I, Flores-Ramos M, Mancilla-Herrera I, Cruz FMC, Hernández-Ruiz J, Diaz GP, et al. Chemokine profile in women with moderate to severe anxiety and depression during pregnancy. BMC Pregnancy Childbirth (2021) 21(1):807.

6. Lim W, Bae H, Bazer FW, Song G. Characterization of C-C motif chemokine ligand 4 in the porcine endometrium during the presence of the maternal-fetal interface. Dev Biol (2018) 441(1):146–58.

7. Chavez B, Kiaris H. Insights on the role of the chemokine CCL8 in pathology. Cell Signal (2025) 134:111951.

8. Nazarinia D, Behzadifard M, Gholampour J, Karimi R, Gholampour M. Eotaxin-1 (CCL11) in neuroinflammatory disorders and possible role in COVID-19 neurologic complications. Acta Neurol Belg (2022) 122(4):865–9.

9. Sharma S, Godbole G, Modi D. Decidual control of trophoblast invasion. Am J Reprod Immunol (2016) 75(3):341–50.

10. Li L, Dai F, Wang L, Sun Y, Mei L, Ran Y, et al. CCL13 and human diseases. Front Immunol (2023) 14:1176639.

11. Laan M, Haljasorg U, Kisand K, Salumets A, Peterson P. Pregnancy-induced thymic involution is associated with suppression of chemokines essential for T-lymphoid progenitor homing. Eur J Immunol (2016) 46(8):2008–17.

12. Wang X, Yip KC, He A, Tang J, Liu S, Yan R, et al. Plasma Olink proteomics identifies CCL20 as a novel predictive and diagnostic inflammatory marker for preeclampsia. J Proteome Res (2022) 21(12):2998–3006.

13. Hua R, Pease JE, Sooranna SR, Viney JM, Nelson SM, Myatt L, et al. Stretch and inflammatory cytokines drive myometrial chemokine expression via NF-κB activation. Endocrinology (2012) 153(1):481–91.

14. Shin S, Kim S, Lee G, Bae J, Kang J, Park J. Profiling cytokines according to the presence or absence of small-for-gestational age using amniotic fluid retrieved during cesarean section from pregnant women with early-onset preeclampsia. J Korean Med Sci (2025) 40(34):e213.

15. Ma C, Liu G, Liu W, Xu W, Li H, Piao S, et al. CXCL1 stimulates decidual angiogenesis via the VEGF-A pathway during the first trimester of pregnancy. Mol Cell Biochem (2021) 476(8):2989–98.

16. Zhang H, Hou L, Li CM, Zhang WY. The chemokine CXCL6 restricts human trophoblast cell migration and invasion by suppressing MMP-2 activity in the first trimester. Hum Reprod (2013) 28(9):2350–62.

17. Accortt E, Mirocha J, Zhang D, Kilpatrick SJ, Libermann T, Karumanchi SA. Perinatal mood and anxiety disorders: biomarker discovery using plasma proteomics. Am J Obstet Gynecol (2023) 229(2):166.e1-166.e16.

18. Brien ME, Boufaied I, Bernard N, Forest JC, Giguere Y, Girard S. Specific inflammatory profile in each pregnancy complication: a comparative study. Am J Reprod Immunol (2020) 84(6):e13316.

19. Kahouadji S, Giguère Y, Lambert S, Forest JC, Bernard N, Blanchon L, et al. CX3CL1/Fractalkine as a biomarker for early pregnancy prediction of preterm premature rupture of membranes. Clin Chem Lab Med (2024) 62(6):1101–8.

20. Cowell W, Colicino E, Lee AG, Bosquet Enlow M, Flom JD, Berin C, et al. Data-driven discovery of mid-pregnancy immune markers associated with maternal lifetime stress: results from an urban pre-birth cohort. Stress (2020) 23(3):349–58.

21. Vilotić A, Nacka-Aleksić M, Pirković A, Bojić-Trbojević Ž, Dekanski D, Jovanović Krivokuća M. IL-6 and IL-8: an overview of their roles in healthy and pathological pregnancies. Int J Mol Sci (2022) 23(23):14574.

22. Duan Y, Wang X, Li M, Zhang C, Li S, Wang R, et al. Association of interleukin-12B gene polymorphisms and mRNA expression with preeclampsia. Eur J Obstet Gynecol Reprod Biol (2022) 269:77–82.

23. Romanowska-Próchnicka K, Felis-Giemza A, Olesińska M, Wojdasiewicz P, Paradowska-Gorycka A, Szukiewicz D. The role of TNF-α and anti-TNF-α agents during preconception, pregnancy, and breastfeeding. Int J Mol Sci (2021) 22(6):2922.

24. Xiao C, Rui Y, Zhou S, Huang Y, Wei Y, Wang Z. TNF-related apoptosis-inducing ligand (TRAIL) promotes trophoblast cell invasion via miR-146a-EGFR/CXCR4 axis: a novel mechanism for preeclampsia? Placenta (2020) 93:8–16.

25. Shahid S, Khalid E, Fatima SS, Khan GM. Evaluation of soluble TNF-like weak inducer of apoptosis (sTWEAK) levels to predict preeclampsia in early weeks of pregnancy. Eur J Obstet Gynecol Reprod Biol (2019) 234:165–70.

26. Simón-Muela I, Llauradó G, Chacón MR, Olona M, Näf S, Maymó-Masip E, et al. Reduced circulating levels of TWEAK are associated with gestational diabetes mellitus. Eur J Clin Invest (2015) 45(1):27–35.

27. Wang W, Parchim NF, Iriyama T, Luo R, Zhao C, Liu C, et al. Excess LIGHT contributes to placental impairment, increased secretion of vasoactive factors, hypertension, and proteinuria in preeclampsia. Hypertension (2014) 63(3):595–606.

28. Yockey LJ, Iwasaki A. Role of Interferons and cytokines in pregnancy and fetal development. Immunity (2018) 49(3):397–412.

29. Jurewicz E, Filipek A. Ca2+- binding proteins of the S100 family in preeclampsia. Placenta (2022) 127:43–51.

30. Cheng SB, Sharma S. Interleukin-10: a pleiotropic regulator in pregnancy. Am J Reprod Immunol (2015) 73(6):487–500.

31. Horvat Mercnik M, Schliefsteiner C, Sanchez-Duffhues G, Wadsack C. TGFβ signalling: a nexus between inflammation, placental health and preeclampsia throughout pregnancy. Hum Reprod Update (2024) 30(4):442–71.

32. Magdoud K, Granados Herbepin V, Messaoudi S, Hizem S, Bouafia N, Almawi WY, et al. Genetic variation in TGFB1 gene and risk of idiopathic recurrent pregnancy loss. Mol Hum Reprod (2013) 19(7):438–43.

33. Zammiti W, Mtiraoui N, Khairi H, Gris JC, Almawi WY, Mahjoub T. Associations between tumor necrosis factor-alpha and lymphotoxin-alpha polymorphisms and idiopathic recurrent miscarriage. Reproduction (2008) 135(3):397–403.

34. Guo X, Yi H, Li TC, Wang Y, Wang H, Chen X. Role of vascular endothelial growth factor (VEGF) in human embryo implantation: clinical implications. Biomolecules (2021) 11(2):253.

35. Dokras A, Gardner LM, Seftor EA, Hendrix MJ. Regulation of human cytotrophoblast morphogenesis by hepatocyte growth factor/scatter factor. Biol Reprod (2001) 65(4):1278–88.

36. Vonderohe C, Guthrie G, Burrin DG. Fibroblast growth factor 19 secretion and function in perinatal development. Amm J Physiol Gastrointest Liver Physiol (2023) 324(3):G190–5.

37. Yang MN, Huang R, Liu X, Xu YJ, Wang WJ, He H, et al. Fibroblast growth factor 19 in gestational diabetes mellitus and fetal growth. Front Endocrinol (2022) 12:805722. doi: 10.3389/fendo.2021.805722.

38. Cao Z, Deng Z, Lu J, Yuan Y. Circulating fibroblast growth factor 21 levels in gestational diabetes mellitus and preeclampsia: a systematic review and meta-analysis. BMC Pregnancy Childbirth (2025) 25:34.

39. Rzewuska-Fijałkowska A, Kwaśniewski W, Gęca T. Role of FGF-19, FGF-21 and FGF-23 in fetal and neonatal growth. J Clin Med (2025) 14(13):4520.

40. Kauma S, Huff T, Krystal G, Ryan J, Takacs P, Turner T. The expression of stem cell factor and its receptor, c-kit in human endometrium and placental tissues during pregnancy. J Clin Endocrinol Metab (1996) 81(3):1261–6.

41. Wang Y, Zhang J, Liang J, Jia L, Niu S, Cheng K, et al. In vivo promotion of primordial follicle activation by stem cell factor treatment in mice with premature ovarian insufficiency and advanced age. Mol Hum Reprod (2023) 29(1):gaac041.

42. Zhou L, Liu J, Zhou M, Xu L. Serum macrophage colony-stimulating factor relates to the severity and the pregnancy outcomes in hypertensive disorders complicating pregnancy. Med Princ Pract (2024) 33(5):462–70.

43. Hamelin-Morrissette J, Dallagi A, Girouard J, Ravelojaona M, Oufqir Y, Vaillancourt C, et al. Leukemia inhibitory factor regulates the activation of inflammatory signals in macrophages and trophoblast cells. Mol Immunol (2020) 120:32–42.

44. Fu T, Zheng HT, Zhang HY, Chen ZC, Li B, Yang ZM. Oncostatin M expression in the mouse uterus during early pregnancy promotes embryo implantation and decidualization. FEBS Lett (2019) 593(15):2040–50.

45. Briana DD, Liosi S, Gourgiotis D, Boutsikou M, Marmarinos A, Baka S, et al. Fetal concentrations of the growth factors TGF-α and TGF-β1 in relation to normal and restricted fetal growth at term. Cytokine (2012) 60(1):157–61.

46. Sahay A, Kale A, Joshi S. Role of neurotrophins in pregnancy and offspring brain development. Neuropeptides (2020) 83:102075.

47. Naruse K, Lash GE, Bulmer JN, Innes BA, Otun HA, Searle RF, et al. The urokinase plasminogen activator (uPA) system in uterine natural killer cells in the placental bed during early pregnancy. Placenta (2009) 30(5):398–404.

48. Maymon E, Romero R, Pacora P, Gervasi MT, Bianco K, Ghezzi F, et al. Evidence for the participation of interstitial collagenase (matrix metalloproteinase 1) in preterm premature rupture of membranes. Am J Obstet Gynecol (2000) 183(4):914–20.

49. Tinsley H, Tryfonos Z, Aziz M, Lagzouli N, Longhurst C, Frick A, et al. Interleukin-1β stimulates matrix metalloproteinase 10 secretion: a possible mechanism in trophoblast-dependent spiral artery remodeling. FASEB J (2025) 39(9):e70597.

50. Atakul N, Atamer Y, Selek Ş, Kılıç B, Koktasoglu F. ST2 and galectin-3 as novel biomarkers for the prediction of future cardiovascular disease risk in preeclampsia. J Obstet Gynaecol (2022) 42(5):1023–9.

51. Bränn E, Edvinsson Å, Rostedt Punga A, Sundström-Poromaa I, Skalkidou A. Inflammatory and anti-inflammatory markers in plasma: from late pregnancy to early postpartum. Sci Rep (2019) 9(1):1863.

52. Sivaraj N, Kusuma BP, Kutikuppala LVS, Rachel KV, Balaga VS, Gundakaram S. Association of TNFSF11 rs2200287 and TNFSF11 rs2148072 gene polymorphisms in preeclampsia. Am J Reprod Immunol (2022) 88(4):e13604.

53. Hong JS, Santolaya-Forgas J, Romero R, Espinoza J, Gonçalves LF, Kim YM, et al. Maternal plasma osteoprotegerin concentration in normal pregnancy. Am J Obstet Gynecol (2005) 93(3 Pt 2):1011–5.

54. Beenen AC, Sauerer T, Schaft N, Dörrie J. Beyond cancer: regulation and function of PD-L1 in health and immune-related diseases. Int J Mol Sci (2022) 23(15):8599.

55. Zhang YH, Tian M, Tang MX, Liu ZZ, Liao AH. Recent insight into the role of the PD-1/PD-L1 pathway in feto-maternal tolerance and pregnancy. Am J Reprod Immunol (2015) 74(3):201–8.

56. Liu X, Aneas I, Sakabe N, Anderson RL, Billstrand C, Paz C, et al. Single cell profiling at the maternal–fetal interface reveals a deficiency of PD-L1+ non-immune cells in human spontaneous preterm labor. Sci Rep (2023) 13(1):7903.

57. He Z, Liu D, Mu Y, Li H. Decoding the maternal–fetal dialogue: NK cell education in reproductive outcomes. Biol Reprod (2025) 113(4):717–29.

58. Gurbanova A, Feyaerts D, Benner M, Meuleman T, van der Hoorn ML, Joosten I, et al. Profiling NK cell receptor repertoire of women with idiopathic recurrent pregnancy loss. J Reprod Immunol (2025) 173:104803.

59. Yoo I, Lee S, Cheon Y, Park TS, Ka H. CD40 ligand and CD40: expression, regulation and function at the maternal-conceptus interface in pigs. Reproduction (2025) 169(3):e240396.

60. Azzam HAG, Abousamra NK, Goda H, El-Shouky R, El-Gilany AH. The expression and concentration of CD40 ligand in normal pregnancy, preeclampsia, and hemolytic anemia, elevated liver enzymes and low platelet count (HELLP) syndrome. Blood Coagul Fibrinolysis (2013) 24(1):71–5.

61. Kövér Á, Lampé R, Szabó K, Tarr T, Papp G. A comprehensive investigation into the distribution of circulating B cell subsets in the third trimester of pregnancy. J Clin Med (2022) 11(11):3006. doi: 10.3390/jcm11113006.

62. Jensen F, Wallukat G, Herse F, Budner O, El-Mousleh T, Costa SD, et al. CD19+CD5+ cells as indicators of preeclampsia. Hypertension (2012) 59(4):861–8.

63. Hardardottir L, Bazzano MV, Glau L, Gattinoni L, Köninger A, Tolosa E, et al. The new old CD8+ T cells in the immune paradox of pregnancy. Front Immunol (2021) 12:765730. doi: 10.3389/fimmu.2021.765730.

64. Wong FTM, Lin C, Cox BJ. Cellular systems biology identifies dynamic trophoblast populations in early human placentas. Placenta (2019) 76:10–8.

65. Viganò P, Somigliana E, Mangioni S, Vignali M, Vignali M, Di Blasio AM. Expression of interleukin-10 and its receptor is up-regulated in early pregnant versus cycling human endometrium. J Clin Endocrinol Metab (2002) 87(12):5730–6.

66. Verma S, Hiby SE, Loke YW, King A. Human decidual natural killer cells express the receptor for and respond to the cytokine interleukin 15. Biol Reprod (2000) 62(4):959–68.

67. Gordon SM. Interleukin-15 in outcomes of pregnancy. In J Mol Sci (2021) 22(20):11094. doi: 10.3390/ijms222011094.

68. Tokmadzić VS, Tsuji Y, Bogović T, Laskarin G, Cupurdija K, Strbo N, et al. IL-18 is present at the maternal-fetal interface and enhances cytotoxic activity of decidual lymphocytes. Am J Reprod Immunol (2002) 48(4):191–200.

69. Eckstrum K, Bany BM. Tumor necrosis factor receptor subfamily 9 (Tnfrsf9) gene is expressed in distinct cell populations in mouse uterus and conceptus during implantation period of pregnancy. Cell Tissue Res (2011) 344(3):567–76.

70. Vukasovic A, Grbesa D, Nikuseva Martic T, Kusec V, Miskovic B, Serman A, et al. Glycosylation pattern and axin expression in normal and IUGR placentae. J Matern Fetal Neonatal Med (2015) 28(5):558–63.

71. Dickinson DP, Thiesse M, Hicks MJ. Expression of type 2 cystatin genes CST1-CST5 in adult human tissues and the developing submandibular gland. DNA Cell Biol (2002) 21(1):47–65.

72. Eiraku M, Hirata Y, Takeshima H, Hirano T, Kengaku M. Delta/notch-like epidermal growth factor (EGF)-related receptor, a novel EGF-like repeat-containing protein targeted to dendrites of developing and adult central nervous system neurons. J Biol Chem (2002) 277(28):25400–7.

73. Li Y, Tang J, Ji X, Hua MM, Liu M, Chang L, et al. Regulation of the mammalian maternal-to-embryonic transition by eukaryotic translation initiation factor 4E. Development (2021) 148(12):dev190793.

74. Tatone C, Di Emidio G, Barbonetti A, Carta G, Luciano AM, Falone S, et al. Sirtuins in gamete biology and reproductive physiology: emerging roles and therapeutic potential in female and male infertility. Hum Reprod Update (2018) 24(3):267–89.

75. Yu Y, An X, Fan D. Histone deacetylase sirtuin 2 enhances viability of trophoblasts through p65-mediated microRNA-146a/ACKR2 axis. Reprod Sci (2021) 28(5):1370–81.

76. Bränn E, Papadopoulos F, Fransson E, White R, Edvinsson Å, Hellgren C, et al. Inflammatory markers in late pregnancy in association with postpartum depression-A nested case-control study. Psychoneuroendocrinology (2017) 79:146–59.

77. Mitra P, Audus KL. Expression and functional activities of selected sulfotransferase isoforms in BeWo cells and primary cytotrophoblast cells. Biochem Pharmacol (2009) 78(12):1475–82.

78. Lestari B, Fukushima T, Utomo RY, Wahyuningsih MSH. Apoptotic and non-apoptotic roles of caspases in placenta physiology and pathology. Placenta (2024) 151:37–47.

79. Lee SJ, Hwang HS, Kim BNR, Kim MA, Lee JW, Park YW, et al. Changes in serum adenosine deaminase activity during normal pregnancy. J Korean Med Sci (2007) 22(4):718–21.
